# Radiopharmaceuticals and theranostics: reshaping cancer diagnosis and therapy with targeted radionuclides

**DOI:** 10.1039/d6ra06959b

**Published:** 2026-10-26

**Authors:** Humaira Zulfiqar, Muhammad Waqas, Ijaz Ali

**Affiliations:** a Department of Chemistry, COMSATS University Islamabad Abbottabad 22044 Pakistan humairazulfiqar774@gmail.com; b Department of Math and Natural Sciences, Gulf University for Science and Technology Hawally 32093 Kuwait

## Abstract

The theranostic paradigm, combining molecular diagnosis and targeted radionuclide therapy in a single platform, has revolutionized precision oncology. Radiopharmaceutical theranostics enables targeted visualization, characterization, and treatment of malignancies through tumor-associated molecular targets, including prostate-specific membrane antigen (PSMA), somatostatin receptors (SSTR), and fibroblast activation protein (FAP). The approvals of ^177^Lu-DOTATATE (Lutathera®) for GEP-NETs and ^177^Lu-PSMA-617 (Pluvicto®) for mCRPC are groundbreaking advances that have triggered exponential growth in the field: the VISION study showed a 38% reduction in the risk of death with ^177^Lu-PSMA-617, and NETTER-1 a 79% reduction in the risk of progression with ^177^Lu-DOTATATE in midgut NETs. Beyond beta emitters, targeted alpha therapy (TAT) with actinium-225, radium-223, and lead-212 is emerging as a powerful strategy to overcome beta-particle resistance, as the high linear energy transfer of alpha particles delivers greater cytotoxicity even in radioresistant tumors. Novel targets such as FAP, CXCR4, HER2, and gastrin-releasing peptide receptor (GRPR) are further diversifying the theranostic armamentarium across malignancies. This review synthesizes the current status of the field, spanning the radiobiology of targeted radionuclide therapy, clinical evidence from pivotal and ongoing trials, alpha-emitting radioisotopes, next-generation radioligands, the growing role of AI-enabled personalized dosimetry, and combination therapies with immunotherapy, PARP inhibitors, and androgen-receptor pathway inhibitors. We also address current challenges heterogeneous target expression, dose-limiting toxicities, radionuclide supply-chain constraints, and the regulatory framework as well as future directions poised to expand the theranostic paradigm to a broader range of cancers.

## Abstract 2

This review traces radiopharmaceutical theranostics from radiobiological mechanism to pivotal trials, highlighting alpha-emitting radionuclides and AI-guided dosimetry as the field's next frontier.

## Graphical abstract

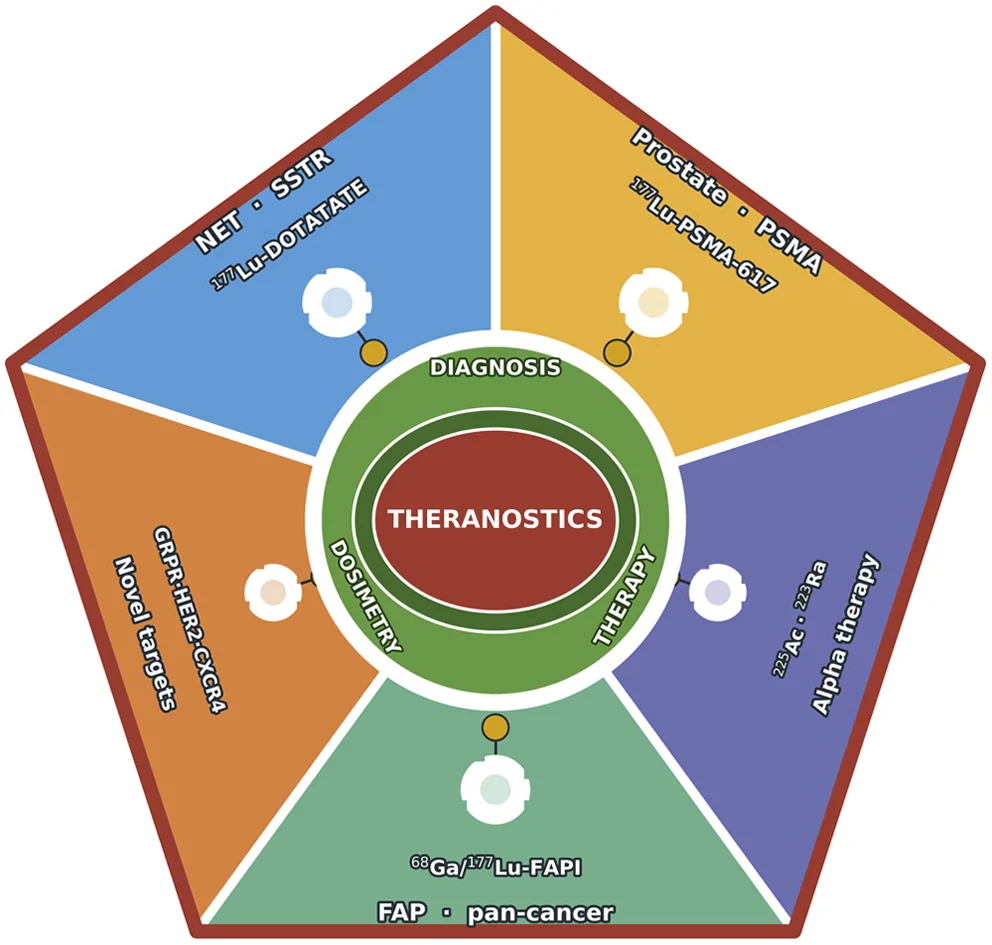

## 1. Introduction

Cancer is the second most common cause of death worldwide, responsible for about 10 million deaths per year, and will cause more than 28 million new cases by 2040.^1^ Even with the dramatic progress in surgical, chemotherapy, targeted, and immunotherapy treatments, malignant diseases remain complex and treatment can become resistant, making new treatment options essential.^2^ The theranostic approach, a combination of targeting and radionuclide therapy, has become a paradigm shift in precision oncology where molecular imaging is combined with targeted radionuclide therapy in one platform.^3^

The theranostic concept itself is not new: the diagnosis and treatment of differentiated thyroid cancer with radioactive iodine (^131^I) has been the archetypal model for over 70 years. Over the past decade, however, advances in molecular biology, radiochemistry, and imaging have driven unprecedented growth: high-affinity radioligands against tumor targets such as PSMA (prostate cancer) and SSTR (neuroendocrine tumors), combined with improved chelation chemistry, have yielded theranostic pairs: diagnostic radionuclides (^68^Ga, ^64^Cu, ^18^F) for PET imaging paired with therapeutic radionuclides (^177^Lu, ^90^Y, ^225^Ac, ^223^Ra) for targeted radionuclide therapy (TRT).^4,5^ This paradigm is outlined in Fig. 1: a PET/SPECT isotope is given for diagnosis and patient selection; a therapeutic alpha-, beta-, or Auger-emitting isotope is then administered over successive cycles at a de-escalating dose as the tumor burden regresses; and follow-up imaging confirms resolution of disease and guides re-assessment.

**Fig. 1:**
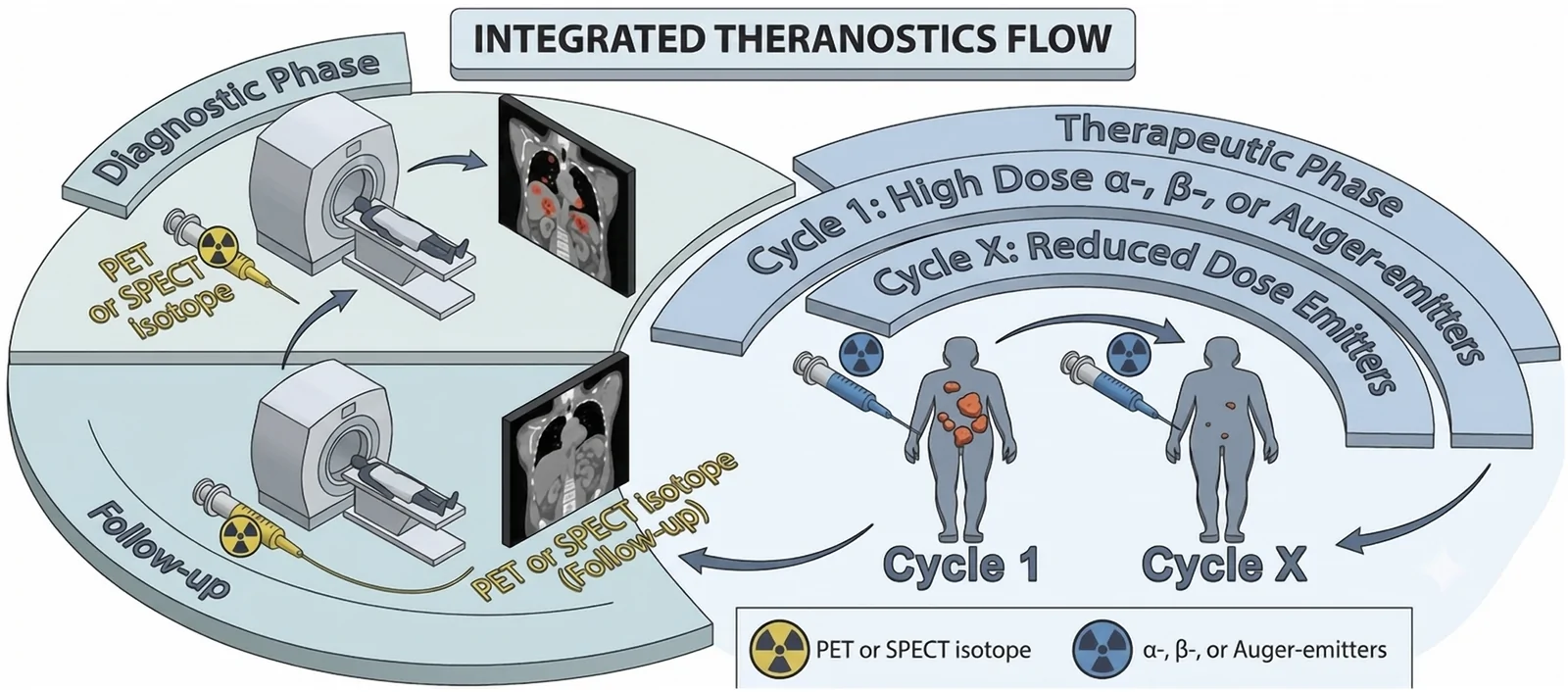
Integrated theranostics flow. In the diagnostic phase, a PET or SPECT isotope is administered and imaging is used to visualize the tumor burden and select the patient for therapy. In the therapeutic phase, the patient receives cyclical radionuclide therapy with alpha-, beta-, or Auger-emitters: an initial high-dose cycle (Cycle 1) targets the full tumor burden, and subsequent cycles (Cycle X) are delivered at a reduced dose as the tumor burden regresses. In the follow-up phase, PET or SPECT imaging is repeated to monitor treatment response, shown here as resolution of the previously tracer-avid lesions, and to guide re-assessment before further treatment cycles.

The same diagnose-and-treat logic extends beyond radiopharmaceuticals to nanomedicine, where multimodal nanoplatforms combine imaging with photodynamic, photothermal or chemodynamic therapy in a single construct.^6^ Radionuclide-based theranostics remains distinguished from these platforms by its reliance on receptor-specific molecular recognition rather than passive or enhanced-permeability-based tumor accumulation.

These scientific advances have been paralleled by an evolving regulatory landscape. Radiopharmaceutical theranostics has now become a clinically proven technique in oncology with the approval of ^68^Ga-DOTATATE (NETSPOT®) and ^68^Ga-PSMA-11 (Locametz®) as companion diagnostic agents in 2016 and 2020, respectively, and ^177^Lu-DOTATATE (Lutathera®) and ^177^Lu-PSMA-617 (Pluvicto®) as therapeutic agents in 2018 and 2022, respectively.^7,8^ This regulatory momentum has, in turn, driven approximately $15 billion in pharmaceutical-industry acquisitions of radiopharmaceutical companies since 2018 (detailed in Section 10.5).^9,10^

In 2025, the global radiopharmaceutical theranostics market is estimated to be worth approximately $2.4 billion and is projected to reach $13.4 billion by 2033, at a compound annual growth rate (CAGR) of approximately 24%. Clinical indications are expanding; new molecular targets such as fibroblast activation protein (FAP) and C-X-C motif chemokine receptor 4 (CXCR4) are being developed; and the introduction of targeted alpha therapy (TAT) with alpha-emitting radionuclides, together with the implementation of artificial intelligence (AI) in personalized dosimetry and treatment optimization, is driving this rapid growth.^11,12^

This review traces the theranostic paradigm from its radiobiological foundations through pivotal-trial evidence, alpha-emitting radionuclides, emerging molecular targets, combination therapy, and AI-enabled dosimetry, before addressing current challenges and future directions; our specific perspective and scope are detailed in Section 11.

Several recent surveys have already summarized individual facets of radiopharmaceutical theranostics: PSMA-targeted radioligand therapy, PRRT in neuroendocrine tumors, or AI-based dosimetry, largely as separate literature. The distinguishing contribution of the present review is to treat radiobiological mechanism, pivotal-trial evidence, emerging radiochemistry (chelator and linker design, Section 2.4), combination pharmacology, and dosimetric/AI infrastructure as a single, mechanistically connected system rather than a set of parallel narratives, and to state explicitly, throughout, which agents are regulatory-approved, which are in registered clinical trials, and which remain preclinical. This integrative framing, developed fully in Section 11, is the review's principal novelty.

Throughout this review, agents are identified by regulatory/development status using three explicit tiers: approved (regulatory-cleared for the stated indication, *e.g.*, ^177^Lu-PSMA-617/Pluvicto®, ^177^Lu-DOTATATE/Lutathera®, ^223^RaCl_2_/Xofigo®), late-stage investigational (in registered Phase II/III trials with reported efficacy/safety data, *e.g.*, ^212^Pb-DOTAMTATE, or ^177^Lu-PSMA-617 in its investigational first-line indication under PSMAddition, Section 3.3), and preclinical/early-phase (first-in-human or animal data only, *e.g.*, the FAP-2286 therapeutic pairing, CXCR4- and CAIX-targeted agents). This tiering is carried into Tables 1–3 and is flagged at first mention of each new agent below.

**Table 1:** Physical properties and clinical applications of important targeting therapeutic radionuclides in oncology^a^

Radionuclide	Emission type	Half-life	Energy (keV)	Tissue range	LET (keV µm^−1^)	Key clinical applications	Regulatory status
^177^Lu	β^−^, γ	6.7 days	497 (β) 208, 113 (γ)	∼2 mm	0.2	mCRPC (PSMA)GEP-NETs (SSTR)	FDA approved (Pluvicto, Lutathera)
^90^Y	β^−^	2.7 days	2280 (β)	∼12 mm	0.2	NHL (CD20) HCC (SIRT)	FDA approved (Zevalin)
^131^I	β^−^, γ	8.0 days	606 (β) 364 (γ)	∼2.4 mm	0.2	DTC (NIS) NHL (CD20)	FDA approved(multiple)
^223^Ra	α	11.4 days	5979 (α)	∼100 µm	80	Bone metastases (CRPC)	FDA approved (Xofigo)
^225^Ac	α (×4)	10.0 days	5830 (α)	40–80 µm	80–100	mCRPC (PSMA) GEP-NETs (SSTR)	Clinical trials (compassionate use)
^212^Pb	β^−^ → ^212^Bi(α)	10.6 h	570 (β) 6090 (α *via*^212^Bi)	∼60 µm (α)	80–100	GEP-NETs (SSTR) (AlphaMedix)	Phase II
^211^At	α	7.2 h	5870 (α)	55–80 µm	97	Brain tumors thyroid cancer	Phase I/II
^161^Tb	β^−^, AE, γ	6.9 days	154 (β) 49 (γ)	∼0.3 mm	0.2 (β) 4–26 (AE)	GEP-NETs (SSTR) prostate (PSMA)	First-in-human studies
^67^Cu	β^−^, γ	2.6 days	577 (β) 185 (γ)	∼2.1 mm	0.2	Various (emerging)	Preclinical/Phase I

a Abbreviations: AE, Auger electron; DTC, differentiated thyroid cancer; CRPC, castration-resistant prostate cancer; GEP-NETs, gastroenteropancreatic neuroendocrine tumors; LET, linear energy transfer; HCC, hepatocellular carcinoma; mCRPC, metastatic castration-resistant prostate cancer; NHL, non-Hodgkin lymphoma; NIS, sodium iodide symporter; SIRT, selective internal radiation therapy.

**Table 2:** Landmark and ongoing clinical trials in radiopharmaceutical theranostics^a^

Trial name	Phase	Agent	Target/indication	Primary endpoint	Key results	Status
VISION (NCT03511664)	III	^177^Lu-PSMA-617 + BSoC *vs.* BSoC	PSMA/mCRPC (post-ARPI and taxane)	OS, rPFS	OS: 15.3 *vs.* 11.3 mo (HR 0.62, *P* < 0.001) rPFS: 8.7 *vs.* 3.4 mo	FDA approved (March 2022)
TheraP (NCT03392428)	II	^177^Lu-PSMA-617 *vs.* cabazitaxel	PSMA/mCRPC (post-docetaxel)	PSA50 response	PSA50: 66% *vs.* 37% (*P* < 0.0001) Fewer grade 3–4 AEs	Completed
PSMAfore (NCT04689828)	III	^177^Lu-PSMA-617 *vs.* ARPI change	PSMA/mCRPC (taxane-naive)	rPFS	rPFS: 12.0 *vs.* 5.6 mo (HR 0.43, *P* < 0.0001)	Completed (final OS 2025)
PSMAddition (NCT04720157)	III	^177^Lu-PSMA-617+ ADT/ARPI vsADT/ARPI	PSMA/mHSPC	rPFS	rPFS HR: 0.72 (*P* = 0.002)	Active (ESMO 2025)
NETTER-1 (NCT01578239)	III	^177^Lu-DOTATATE *vs.* octreotide LAR 60 mg	SSTR2/Midgut NETs	PFS	PFS: NR *vs.* 8.4 mo (HR 0.21, *P* < 0.001)	FDA approved (Jan 2018)
NETTER-2 (NCT03972488)	III	^177^Lu-DOTATATE + octreotide *vs.* octreotide	SSTR2/GEP-NETs (1st line, G2-3)	PFS	PFS: 22.8 *vs.* 8.5 mo (HR 0.27)	Completed (2024)
ALSYMPCA (NCT00699751)	III	^223^Ra-dichloride *vs.* placebo	Bone/CRPC with bone mets	OS	OS: 14.9 *vs.* 11.3 mo (HR 0.70, *P* = 0.002)	FDA approved (May 2013)
AlphaMedix phase 2	II	^212^Pb-DOTAMTATE	SSTR2/GEP-NETs	ORR	Met all primary efficacy endpoints in both cohorts	Phase II completed (ESMO 2025)
TATCIST (NCT05219500)	II	^225^Ac-PSMA-I&T	PSMA/mCRPC	PSA50 response	Pending	Active
LuMIERE	I/II	^177^Lu-FAP-2286	FAP/multiple solid tumors	Safety, ORR	Enrollment ongoing	Active

a Abbreviations: ADT, androgen deprivation therapy; ARPI, androgen receptor pathway inhibitor; BSoC, best standard of care; CRPC, castration-resistant prostate cancer; FAP, fibroblast activation protein; GEP-NETs, gastroenteropancreatic neuroendocrine tumors; mCRPC, metastatic castration-resistant prostate cancer; mHSPC, metastatic hormone-sensitive prostate cancer; NR, not reached; ORR, objective response rate; OS, overall survival; PFS, progression-free survival; rPFS, radiographic progression-free survival.

**Table 3:** New molecular targets for radiopharmaceutical theranostics in oncology^a^

Target	Expression pattern	Diagnostic agent	Therapeutic agent	Key cancer types	Development stage	Key limitations//data gaps
FAP	CAFs in >90% epithelial carcinomas	^68^Ga-FAPI-46 ^68^Ga-FAP-2286	^177^Lu-FAP-2286 ^225^Ac-FAPI	Pancreatic, CRC, breast, sarcoma, cholangiocarcinoma	Phase I/II (LuMIERE)	Rapid renal clearance limits tumour retention of first-generation FAPI tracers; validated TBR/dosimetry data are limited to small single-arm cohorts, with no randomized comparison against SSTR/PSMA-RLT.
CXCR4	Hematological malignancies, select solid tumors	^68^Ga-Pentixafor	^177^Lu/^90^Y-Pentixather	Multiple myeloma, AML, NHL, CLL	Compassionate use/Phase I	Experience limited to compassionate-use and small phase I cohorts (*n* < 30 in most series); on-target myelosuppression from CXCR4 expression on normal haematopoietic progenitors constrains achievable activity
GRPR	Prostate, breast, SCLC, GIST	^68^Ga-RM2 ^68^Ga-NeoBOMB1	^177^Lu-RM2 ^177^Lu-NeoBOMB1	Prostate (PSMA-negative), breast	Phase I/II	Early agonist trials caused physiological side effects, prompting a shift to antagonists (RM2, NeoBOMB1) whose comparative dosimetry against PSMA agents is not yet established in randomized data
HER2	15–20% breast, gastric, esophageal	^68^Ga/^89^Zr-trastuzumab	^225^Ac-pretargeted RIT	Breast, gastric	Preclinical/Phase I	Antibody vectors confer long blood residence and elevated marrow/liver dose; pretargeting to shorten effective half-life remains preclinical, with no clinical TBR or therapeutic dosimetry data yet available
CAIX	Clear cell RCC (>95%)	^89^Zr-girentuximab	^177^Lu-girentuximab	Clear cell renal cell carcinoma	Phase 1b/2–2 (STARLITE)	STARLITE-1 and STARLITE-2 are both single-arm, non-randomized trials, and neither has yet reported comparative efficacy data against standard of care
Mesothelin	Mesothelioma, ovarian, pancreatic	^89^Zr-anetumab	^227^Th-anetumab	Mesothelioma, ovarian, pancreatic	Phase I	Thorium-227 dosimetry in humans is preliminary; daughter-nuclide redistribution from ^227^Th (analogous to the ^225^Ac problem, Section 5.3) is a class-level limitation not yet fully characterized here
DLL3	SCLC, large cell NEC	In development	^225^Ac-SC16 (monoclonal antibody)	SCLC, neuroendocrine carcinoma	Preclinical/Phase I	Direct radiolabeling of a full-length monoclonal antibody confers long blood residence and marrow dose; reported data remain preclinical, with no published human dosimetry or TBR values

a Abbreviations: AML, acute myeloid leukemia; CAFs, cancer-associated fibroblasts; CAIX, carbonic anhydrase IX; CLL, chronic lymphocytic leukemia; CRC, colorectal cancer; DLL3, delta-like ligand 3; FAP, fibroblast activation protein; GIST, gastrointestinal stromal tumor; GRPR, gastrin-releasing peptide receptor; NEC, neuroendocrine carcinoma; NHL, non-Hodgkin lymphoma; RCC, renal cell carcinoma; RIT, radioimmunotherapy; SCLC, small cell lung cancer.

To make this integration explicit, the running order of Sections 3–6 is organized around a single conceptual axis: molecular target expression → ligand/chelator chemistry → radionuclide physics → dosimetric consequence → clinical outcome. Each target-specific section (PSMA, SSTR, FAP, CXCR4, GRPR, HER2) is structured to answer the same four questions: why this target, why this radionuclide pairing, what the chemistry does mechanistically, and what the trial data show, rather than being presented as an independent case study; cross-references between sections (*e.g.*, the chelator chemistry introduced in Section 2.4 and revisited for the Ac-225 daughter problem in Section 5.3) reinforce this framework.

## 2. Radiobiological principles of targeted radionuclide therapy

### 2.1 Mechanisms of radiation-induced cell death

Targeted radionuclide therapy (TRT) exploits the cytotoxic potential of ionizing radiation delivered directly to tumor cells *via* receptor-mediated binding of a radiolabeled ligand. Unlike external beam radiotherapy (EBRT), which irradiates from an external source, TRT delivers continuous systemic radiation to primary and metastatic lesions including micrometastases below the resolution of conventional imaging.^13,14^ Cell killing occurs mainly through DNA double-strand breaks (DSBs), which, if unrepaired or misrepaired, trigger apoptosis, mitotic catastrophe, necrosis, or senescence.^15^ High-LET alpha particles (50–230 keV µm^−1^) deposit energy within 50–100 µm (Sgouros *et al.*, 2010), concentrating cytotoxicity within the tumor, whereas low-LET beta particles (∼0.2 keV µm^−1^) travel 2–12 mm, spanning the clinically used range from ^177^Lu (∼2 mm; Müller *et al.*, 2012) to ^90^Y (∼12 mm; Eiber *et al.*, 2017), and irradiate healthy tissue as well (Fig. 2).

**Fig. 2:**
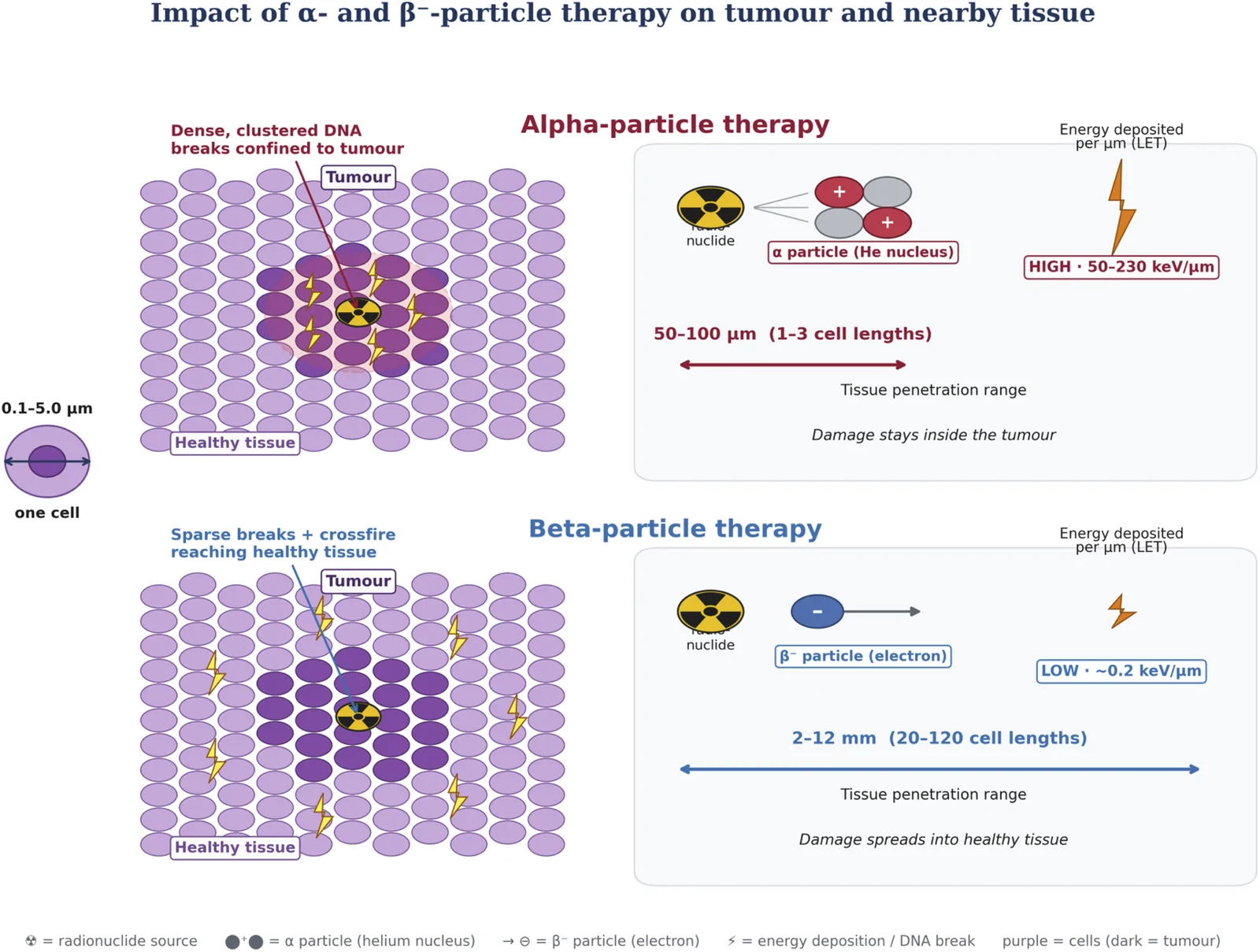
The effects of alpha and beta radiations on tumor and surrounding tissues. The tissue penetration range of alpha particles is 50–100 µm (1–3 cell lengths) and their linear energy transfer (LET) is high (50–230 keV µm^−1^) which results in clustered double-strand DNA breaks limited to the tumor. The beta particles are low LET (0.2 keV µm^−1^) and penetrate 2–12 mm (20–120 cell lengths) into the tissue, impacting a wider tissue volume, including healthy tissue.

Several physical and biological parameters affect the biological effectiveness of TRT, including LET, dose rate, subcellular localization of the radionuclide, and the intrinsic radiosensitivity of target cells.^16^ The longer tissue range of beta-emitters such as ^177^Lu and ^90^Y allows a ‘crossfire’ effect, in which radiation from radiolabeled cells kills neighboring unlabeled cells within the tumor microenvironment a useful property when target expression is heterogeneous.^17^

Alpha particles, emitted by radionuclides such as ^225^Ac, ^223^Ra, ^212^Pb, and ^211^At, produce a much higher ionization density along their short path, causing complex DSBs that cellular repair enzymes poorly resolve.^18,19^ Consequently their relative biological effectiveness (RBE) is 3–7 times that of beta particles, and their cytotoxicity is independent of cell-cycle phase, oxygen status, or dose rate making them effective against radioresistant hypoxic and quiescent tumor cells.^20,21^

### 2.2 Crossfire, bystander, and abscopal effects

Beyond direct DNA damage, TRT produces indirect biological effects linked to its therapeutic activity. The crossfire effect (above) extends the cytotoxic reach of beta-emitters beyond targeted cells, which is valuable against heterogeneous target expression. The bystander effect occurs when damaged cells release stress signals *via* gap junctions and soluble mediators that induce DNA damage, chromosomal instability, and apoptosis in neighboring, non-targeted cells.^22^

The abscopal effect a systemic antitumor immune response induced by local radiation has drawn particular attention in combination with immune checkpoint inhibitors (ICIs). Radiation-induced immunogenic cell death releases tumor-associated antigens, DAMPs, and pro-inflammatory cytokines that activate dendritic cells and tumor-specific T-cell responses.^23^ Because TRT irradiates multiple metastatic deposits simultaneously, unlike single-site EBRT, it may amplify this immunogenic effect and convert ‘cold’ tumors to ‘hot’ one rationale behind the TRT–immunotherapy combination trials discussed in Section 7.

These three indirect phenomena are frequently conflated but are mechanistically and spatially distinct. The crossfire effect is a purely physical consequence of particle range: it kills antigen-negative or under-expressing cells within the emission radius of a bound radionuclide (millimeters for β^−^-emitters such as ^177^Lu and ^90^Y, sub-millimeter for α-emitters) and requires no immune involvement. The bystander effect operates at a shorter, cell-to-cell scale, mediated by gap-junction signaling, secreted cytokines (TGF-β, IL-6) and reactive oxygen species released by directly irradiated cells, producing DNA damage in neighboring unirradiated cells that need not even lie within the physical particle range. The abscopal effect operates at whole-organism scale and is immune-mediated, requiring antigen release, dendritic-cell cross-presentation and a systemic cytotoxic T-cell response, which is why it is reported far less consistently than crossfire or bystander killing and, clinically, is almost always documented alongside concurrent immune checkpoint blockade (Section 7.1) rather than after TRT alone. Distinguishing the three matters practically: crossfire is what allows PRRT and PSMA-RLT to work in heterogeneous, non-uniformly-expressing tumors, whereas the abscopal effect is the mechanistic rationale for the RLT–ICI combination trials discussed in Section 7.

### 2.3 Radionuclide selection: physical and chemical considerations

Radionuclide choice for TRT depends on physical decay properties (half-life, emission type, particle energy, tissue range), chemical properties (coordination chemistry, labeling efficiency, *in vivo* stability), and clinical factors (availability, cost, waste management).^24^ Ideally, the radionuclide's half-life should match the carrier molecule's pharmacokinetics, its LET and range should suit tumor size, and it should be chelatable or covalently linkable to the carrier.^25^

Lutetium-177 (^177^Lu) is the most widely used therapeutic radionuclide, combining medium-energy beta decay (Eβ_max_ = 497 keV, tissue range ∼2 mm) with low-energy gamma photons (208, 113 keV) suitable for post-therapy SPECT/CT dosimetry.^26^ Yttrium-90 (^90^Y) emits higher-energy beta particles (Eβ_max_ = 2.28 MeV, range ∼12 mm) with no imageable gamma, favoring larger tumors but carrying greater renal-toxicity risk.^27^ Among alpha emitters, actinium-225 (^225^Ac; *t*_1/2_ = 10 days) is attractive for producing four alpha particles per decay,^28^ though its daughter radionuclides (^221^Fr, ^217^At, ^213^Bi) cause dosimetric off-target toxicity, particularly to kidneys and salivary glands.^29^

The choice of which therapeutic radionuclide to pair with a given diagnostic isotope follows directly from these physical parameters rather than from convenience. Slowly proliferating, well-vascularized tumors with homogeneous receptor expression (*e.g.*, well-differentiated NETs) are well matched to the millimeter-range, lower-LET β^−^-emitters ^177^Lu and ^90^Y, whose longer path length provides crossfire across heterogeneous receptor density without requiring precise subcellular targeting. Rapidly proliferating, micrometastatic or minimal-residual disease, by contrast, is the rationale most often invoked for α-emitters (^225^Ac, ^223^Ra, ^212^Pb, ^211^At): their short range confines the high-LET dose to single-cell or few-cell volumes, which is dosimetrically advantageous when target density is low or disease burden is sub-radiographic, but is a liability when receptor expression is markedly heterogeneous, since neighboring antigen-negative clonal populations receive negligible crossfire dose. Radionuclide half-life must in turn match the biological half-life of the vector: small peptides and PSMA inhibitors clear renally within hours and are typically paired with radionuclides of hours-to-days half-life (^68^Ga, ^177^Lu, ^212^Pb), whereas intact antibodies with multi-day circulation are better matched to longer-lived isotopes (^89^Zr, *t*_1/2_ = 78.4 h, for immunoPET; ^225^Ac, *t*_1/2_ = 10 days, for radioimmunotherapy) to avoid radionuclide decay before tumor accumulation peaks.

### 2.4 Radiochemistry: chelator design, radiolabeling and metabolic fate

Beyond receptor recognition, the choice of chelator dictates whether a radiometal is retained stably *in vivo* or leaches to bone and marrow as free ion, and must be matched to the coordination preferences of the specific radiometal used at each stage of the theranostic pair. Ga^3+^ (ionic radius ∼62 pm) is efficiently chelated by the hexadentate macrocycle NOTA or the octadentate DOTA at room temperature within minutes; the substantially larger, softer Lu^3+^ and Y^3+^ (ionic radius ∼86–90 pm) used for β^−^-therapy require the higher denticity and larger cavity of DOTA, typically labeled at 90–95 °C for 15–30 min to achieve radiochemical purity >95% (Benešová *et al.*, 2015). Ac^3+^ is larger still (ionic radius ∼112 pm) and is poorly accommodated by DOTA's cavity,^30^ which is the root chemical cause of ^225^Ac-DOTA conjugates' *in vivo* instability and consequent daughter-nuclide release (Section 5.3); this has driven development of larger 18-membered macrocyclic chelators purpose-built for Ac^3+^, notably macropa^31^ and the crown-ether/py4pa families, which show markedly improved kinetic inertness against transchelation to serum proteins *in vitro*.

Linker chemistry between the targeting vector and the chelator is not innocent: it sets lipophilicity, plasma-protein binding, renal *versus* hepatobiliary clearance route, and, for internalizing receptors, the rate and extent of lysosomal residualization of the radiometal after receptor-mediated endocytosis. PSMA-617 and PSMA-I&T differ principally in linker composition, and this measurably shifts salivary-gland uptake and tumor residence time between the two otherwise similar agents. Radiolabeling is generally performed at high molar activity (low carrier, ideally >20–50 GBq µmol^−1^ for peptides) to avoid saturating the finite receptor population with non-radioactive (cold) ligand, since co-administered unlabeled peptide competes for the same binding sites and can reduce tumor uptake of the radiolabeled fraction. Radiochemical purity (the fraction of activity present as the intact labeled species, verified by radio-HPLC or radio-iTLC) is typically required to exceed 95% for clinical release, and *in vivo* metabolic stability, resistance to peptidase cleavage of the linker or transchelation of the metal to transferrin/serum proteins, determines whether the biodistribution measured shortly after injection still reflects intact tracer at the time of dosimetric imaging days later.

Table 1 summarizes the physical/chemical properties, clinical uses, and regulatory status of the most relevant therapeutic radionuclides ranging from the workhorse, millimeter-range, low-LET β^−^-emitters ^131^I and ^177^Lu to the short-range (50–100 µm), high-LET (80–100 keV µm^−1^) α-emitters ^211^At, ^212^Pb, ^223^Ra, and ^225^Ac better suited to small tumor deposits or minimal residual disease, plus emerging agents such as ^161^Tb and ^67^Cu in early clinical development. Notably, only ^177^Lu, ^90^Y, ^131^I, and ^223^Ra currently hold full regulatory approval, reflecting the supply, production, and dosimetry challenges that still constrain widespread α-emitter adoption.

## 3. PSMA-targeted radioligand therapy in prostate cancer

### 3.1 Biological rationale and target biology

Prostate-specific membrane antigen (PSMA) also known as glutamate carboxypeptidase II or folate hydrolase 1 is a type II transmembrane glycoprotein whose expression on prostate cancer cells rises with tumor grade, stage, androgen independence, and metastatic progression,^32^ reaching 100-1000-fold higher levels than normal prostatic epithelium, especially in castration-resistant and metastatic disease.^33^ Ligand binding triggers constitutive PSMA internalization *via* clathrin-coated pits, increasing intracellular radiation dose deposition.^34^

The PSMA theranostic paradigm rests on small-molecule PSMA-enzyme inhibitors radiolabeled with ^68^Ga or ^18^F for PET/CT (companion diagnostic) and ^177^Lu or ^225^Ac for therapy.^35^ PSMA PET/CT outperforms conventional imaging (CT, bone scan, ^68^Ga-choline PET) in detecting primary and recurrent prostate cancer, with >90% sensitivity and >95% specificity.^36,37^

### 3.2 The VISION trial: establishing the standard of care

VISION (NCT03511664), a landmark international randomized phase III trial, established ^177^Lu-PSMA-617 as a new standard of care for PSMA-positive mCRPC progressing after ARPIs and taxane chemotherapy.^38^ Its 831 patients were randomized 2 : 1 to ^177^Lu-PSMA-617 (7.4 GBq every 6 weeks, up to 6 cycles) plus best standard of care (BSoC), or BSoC alone.

Median OS was significantly longer with ^177^Lu-PSMA-617 plus BSoC than BSoC alone (15.3 *vs.* 11.3 months; HR 0.62; 95% CI 0.52–0.74; *P* < 0.001—a 38% mortality reduction), as was rPFS (60% reduction in risk of progression/death; median 8.7 *vs.* 3.4 months; HR 0.40; 99.2% CI 0.29–0.57; *P* < 0.001).^39^ Secondary endpoints also favored ^177^Lu-PSMA-617: time to first symptomatic skeletal event (11.5 *vs.* 6.8 months; HR 0.50), ORR (29.8% *vs.* 1.7%), and PSA50 response (46% *vs.* 7%).^39^

The safety profile was manageable: the most common grade 3–4 events were anemia (12.9%), thrombocytopenia (7.9%), lymphopenia (7.8%), and fatigue (5.9%), while 39% of patients experienced mostly grade 1–2 xerostomia from salivary-gland PSMA expression. No treatment-related deaths occurred, and quality-of-life measures (pain, physical functioning, fatigue) improved relative to BSoC.^40^ These results led to FDA approval of ^177^Lu-PSMA-617 (Pluvicto®) in March 2022 for PSMA-positive mCRPC after ARPI and taxane chemotherapy.^41^

Critically, VISION enrolled a molecularly pre-selected population (PSMA-PET-positive, taxane- and ARPI-exposed) that represents a relatively favorable-biology subset of mCRPC; the trial did not mandate a PSMA-PET-negative comparator arm, so the incremental benefit in patients with heterogeneous or low PSMA expression, roughly 10–30% of screened patients who were PET-ineligible (Section 9.1), cannot be inferred from these data, and cross-trial comparison with PSMAfore is confounded by differences in prior-therapy exposure and control arm (best standard of care *vs.* ARPI switch).

### 3.3 Moving to earlier lines of therapy: PSMAfore and PSMAddition trials

PSMAfore (NCT04689828), a VISION follow-on trial, tested ^177^Lu-PSMA-617 in taxane-naive PSMA-positive mCRPC patients who had failed one prior ARPI. In this randomized phase III trial, 468 patients received ^177^Lu-PSMA-617 or an ARPI switch (enzalutamide↔abiraterone) 1 : 1.^42^ rPFS, the primary endpoint, improved significantly (median 12.0 *vs.* 5.6 months; HR 0.43; 95% CI 0.33–0.54; *P* < 0.0001); final OS (2025) showed no significant difference (HR 0.95), likely reflecting the 84% crossover rate from control to ^177^Lu-PSMA-617.^43^ Safety was comparable to VISION with no new signals.

The PSMAddition trial (NCT04720157) is exploring the addition of ^177^Lu-PSMA-617 to standard-of-care ADT + ARPI in newly diagnosed mHSPC the first time RLT has been evaluated in a first-line disease setting. In this randomized 1 : 1 phase III trial of 1144 patients, preliminary ESMO 2025 data showed the ^177^Lu-PSMA-617 arm had significantly prolonged rPFS (HR 0.72; 95% CI 0.58–0.90; *P* = 0.002), with 86% completing all 6 cycles,^44^ suggesting RLT could move into first-line treatment for a much larger metastatic prostate cancer population.

### 3.4 Targeted alpha therapy with ^225^Ac-PSMA

Targeted alpha therapy (TAT) with ^225^Ac-PSMA-617 or ^225^Ac-PSMA-I&T is a promising salvage option after progression on ^177^Lu-PSMA-617. Compassionate-use programs report >50% PSA reduction in roughly 60–70% of such patients,^45^ with the higher LET and RBE of alpha particles overcoming the relative radioresistance that limits beta-particle therapy.

However, ^225^Ac-PSMA therapy carries significant xerostomia risk (up to 70–87% of patients), which can limit dosing and quality of life.^46^ Mitigation strategies include PSMA-binding blockers (*e.g.*, 2-PMPA), salivary gland cooling, and lower-uptake PSMA ligands. The phase II TATCIST trial (NCT05219500) is evaluating ^225^Ac-PSMA-I&T in mCRPC, with prospective efficacy/safety data expected in 2026.^47^

Two next-generation PSMA ligands developed specifically to reduce this xerostomia burden further illustrate how linker/chelator redesign translates into clinical benefit. PSMA-62 (paired with ^225^Ac as LY4181530/PNT2001) was engineered for improved cellular internalization relative to PSMA-617 and PSMA-I&T, and is now being evaluated in the multicenter ACCEL Phase Ia/Ib/II trial (NCT06229366) in oligometastatic hormone-sensitive and metastatic castration-resistant disease (Saleh *et al.*, 2025). ^225^Ac-PSMA-CY313 takes a different chemical route to the same goal, combining a highly hydrophilic scaffold (log *P* = −3.7) with high PSMA affinity (Ki = 17.1 nM) to reduce salivary and lacrimal retention while preserving tumor-to-muscle ratios superior to PSMA-617 in early comparative data; a first-in-human study in 16 mCRPC patients reported predominantly low-grade, reversible xerostomia alongside a 37.5% PSA50 response rate (Zhang *et al.*, 2026). Both agents support the broader chemical principle that xerostomia in ^225^Ac-PSMA therapy is not an immutable biological ceiling but a linker/lipophilicity–tunable parameter.

Table 2 summarizes the pivotal and ongoing trials that established radiopharmaceutical theranostics as standard of care: VISION for ^177^Lu-PSMA-617, NETTER-1/NETTER-2 for ^177^Lu-DOTATATE in NETs, and ALSYMPCA for ^223^Ra in bone-metastatic CRPC, alongside expansion trials (TheraP, PSMAfore, PSMAddition) and a maturing alpha-therapy pipeline extending beyond prostate/neuroendocrine indications (AlphaMedix, TATCIST, LuMIERE).

## 4. Peptide receptor radionuclide therapy for neuroendocrine tumors

### 4.1 Somatostatin receptor biology and the theranostic paradigm

Neuroendocrine tumors (NETs) are a heterogeneous group arising from the diffuse neuroendocrine system, most commonly in the gastrointestinal tract and lungs. Well-differentiated NETs characteristically overexpress somatostatin receptors, with SSTR2 present in >80% of gastroenteropancreatic NETs (GEP-NETs).^48^ This overexpression is exploited both diagnostically (^68^Ga-DOTATATE/DOTATOC PET/CT) and therapeutically (radiolabeled somatostatin analogs), making SSTR-targeted therapy the prototype of peptide receptor radionuclide therapy (PRRT).^49^

The Krenning score, a semiquantitative measure of SSTR expression on ^68^Ga-DOTA-peptide PET/CT, guides patient selection: tumors with uptake exceeding liver background (score ≥3) are considered good PRRT candidates (Krenning, 1993) a clear example of the personalized-medicine logic inherent to theranostics.

### 4.2 The NETTER-1 trial: establishing PRRT as standard of care

The NETTER-1 trial (NCT01578239), a landmark multicenter randomized phase III trial, established ^177^Lu-DOTATATE as standard of care for progressive, SSTR-positive, well-differentiated midgut NETs. 229 patients were randomized 1 : 1 to ^177^Lu-DOTATATE (7.4 GBq every 8 weeks, 4 cycles) plus octreotide LAR 30 mg, or high-dose octreotide LAR 60 mg alone. PFS, the primary endpoint, improved dramatically with ^177^Lu-DOTATATE (median not reached *vs.* 8.4 months; HR 0.21; 95% CI 0.13–0.33; *P* < 0.001 a 79% reduction in progression risk). Results are shown in Fig. 3: the PFS benefit (panel A) and the corresponding 79% reduction in the risk of progression or death (panel B) (Strosberg *et al.*, 2017).

**Fig. 3:**
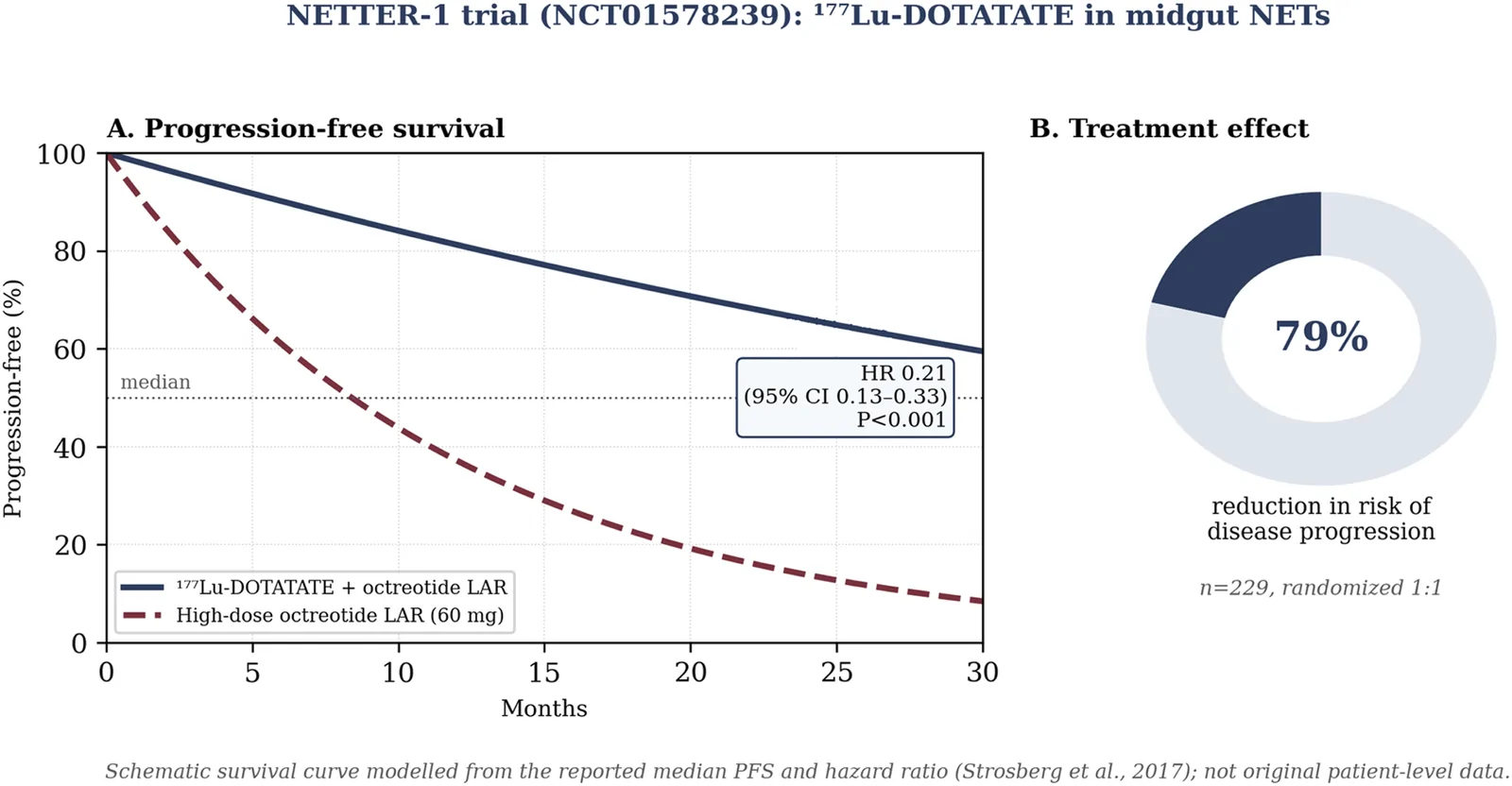
Outcomes of the NETTER-1 trial (NCT01578239): ^177^Lu-DOTATATE in advanced midgut neuroendocrine tumors. (A) Progression-free survival ^177^Lu-DOTATATE plus octreotide LAR markedly prolonged PFS compared with high-dose octreotide LAR (60 mg); the ^177^Lu-DOTATATE median was not reached *versus* approximately 8 months in the control arm (HR 0.21, 95% CI 0.13–0.33; *P* < 0.001). (B) Treatment effect this corresponds to a 79% reduction in the risk of disease progression or death (*n* = 229, randomized 1 : 1). Survival curves are schematic, modelled from the reported median PFS and hazard ratio (Strosberg *et al.*, 2017), and do not represent original patient-level data.

20 months PFS probability was 65.2% with PRRT *vs.* 10.8% with control. Long-term follow-up showed a non-significant OS trend favoring ^177^Lu-DOTATATE (median 48.0 *vs.* 36.3 months; HR 0.84), likely reflecting crossover design. ORR improved to 18% *vs.* 3% (*P* < 0.001), with significant quality-of-life gains. Grade 3–4 hematologic toxicities were lymphopenia (9%), thrombocytopenia (2%), and anemia (1%); renal toxicity was rare (<1%). These results led to FDA approval of ^177^Lu-DOTATATE (Lutathera®) in January 2018 for SSTR-positive GEP-NETs.^50^

The absence of a statistically significant overall-survival benefit despite the large PFS effect is attributable in part to the trial's crossover design, which allowed control-arm patients to receive PRRT after progression and therefore diluted any survival difference; this is a recurring limitation across PRRT trials and should temper cross-trial ranking of PFS effect sizes as a proxy for long-term benefit.

### 4.3 The NETTER-2 trial: first-line PRRT

The NETTER-2 trial (NCT03972488) extended this to the first-line setting in newly diagnosed, advanced, well-differentiated (grade 2–3) SSTR-positive GEP-NETs, comparing ^177^Lu-DOTATATE plus long-acting octreotide to high-dose octreotide alone. The primary endpoint was met, with median PFS of 22.8 *vs.* 8.5 months (HR 0.27; *P* < 0.0001),^51^ suggesting PRRT could become a first-line option for high-grade GEP-NETs rather than a salvage strategy.^52^

### 4.4 Alpha-PRRT and next-generation approaches

Resistance to conventional beta-PRRT has prompted clinical investigation of alpha-emitting PRRT with ^212^Pb-DOTAMTATE (AlphaMedix™) and ^225^Ac-DOTATATE. In a phase II trial evaluating ^212^Pb-DOTAMTATE in PRRT-naive (*n* = 35) and PRRT-exposed (*n* = 26) patients with unresectable/metastatic SSTR-positive GEP-NETs, data presented at ESMO 2025 showed all primary efficacy endpoints met. Per investigator assessment (RECIST 1.1), the confirmed objective response rate (ORR) was 54.3% (19/35 evaluable patients; 95% CI, 38.2–69.5%) in the PRRT-naive cohort, and 34.6% (9/26 evaluable patients, all partial responses) in the PRRT-exposed cohort, with a disease control rate of 96.2% (95% CI, 80.4–99.9%) and 18 months progression-free/overall survival of 82.6%/85.1% in the PRRT-exposed cohort, reinforcing the first-in-class potential of ^212^Pb-based TAT. The short half-life of ^212^Pb (10.6 h *vs.*^225^Ac's 10 days) also limits daughter-redistribution toxicity and can be produced on-demand from a ^224^Ra generator, easing supply constraints.^53^

SSTR antagonists offer a promising alternative to conventional agonists (DOTATATE, DOTATOC). Antagonists such as DOTA-JR11 (satoreotide tetraxetan) bind more receptor sites on the cell surface, yielding 1.7- to 10.6-fold higher preclinical tumor uptake than ^177^Lu-DOTATATE; clinical trials assessing therapeutic efficacy are ongoing.^54^

## 5. Targeted alpha therapy: the next frontier

### 5.1 Rationale for alpha-emitting radionuclides

Targeted alpha therapy (TAT) is among the most important advances in radiopharmaceutical theranostics. Alpha particles deposit 100 to 1000 times more energy per unit path length than beta particles, producing 5–10 DSBs per traversal *versus* 0.5–1 for beta particles. This dense ionization renders the damage nearly irreparable by homologous recombination or non-homologous end joining, causing cell death at very low absorbed doses.

This short range (50–100 µm) is a double-edged sword: it concentrates the cytotoxic dose within a few cell diameters of the target, sparing surrounding tissue and, in theory, improving the therapeutic index over beta-emitters, but the limited crossfire also means target heterogeneity in bulky tumors can leave non-targeted cells subtherapeutically dosed.^55^

### 5.2 Radium-223 dichloride: the first approved alpha-emitter

The first alpha emitter approved for cancer treatment was radium-223 dichloride (^223^RaCl_2_, Xofigo®). As a calcium mimetic, ^223^Ra selectively deposits in osteoblastic bone metastases, incorporating into the hydroxyapatite matrix to deliver highly localized alpha radiation.^56^

The pivotal ALSYMPCA trial (NCT00699751) enrolled 921 CRPC patients with symptomatic bone metastases who had received, were ineligible for, or declined docetaxel, randomizing them to six 4-weekly ^223^Ra injections (55 kBq kg^−1^) or placebo. OS was significantly longer with ^223^Ra (14.9 *vs.* 11.3 months; HR 0.70; 95% CI 0.58–0.83; *P* = 0.002 a 30% mortality reduction), as was time to first symptomatic skeletal event (15.6 *vs.* 9.8 months; HR 0.66; *P* < 0.001). Myelosuppression was low, reflecting alpha particles' short range and bone-marrow sparing, making ^223^Ra the first and still only approved alpha-emitting radiopharmaceutical in oncology. More recently, PEACE-3 showed that adding ^223^Ra to enzalutamide in mCRPC with bone metastases confers a substantial survival advantage, suggesting alpha-emitter combination therapy may be even more effective.^57^

ALSYMPCA's population was, by design, restricted to symptomatic bone-only or bone-predominant CRPC without bulky visceral or nodal disease; ^223^Ra's survival benefit therefore cannot be extrapolated to patients with visceral metastases, and the trial predates routine PSMA-PET staging, so some enrolled patients would today be re-classified and offered PSMA-RLT instead, a caveat relevant to interpreting ^223^Ra's current, narrower place in the treatment sequence.

### 5.3 Actinium-225: promise and challenges

Actinium-225 (^225^Ac) is the most clinically studied alpha emitter after ^223^Ra. Its 10 days half-life suits small molecules and peptides, and its decay produces four net alpha particles, each capable of lethal DNA damage. Kratochwil *et al.*'s pioneering study showed ^225^Ac-PSMA-617 achieved important biochemical responses in mCRPC after ^177^Lu-PSMA-617 failure, with >50% PSA decline in ∼63% of patients and complete biochemical response in a subset (Feuerecker *et al.*, 2021).

Clinical adoption nonetheless faces obstacles. Global ^225^Ac supply remains very limited: production depends on ^229^Th stockpiles at Oak Ridge National Laboratory and the Institute for Transuranium Elements, sufficient for only ∼1000 patient treatments annually. Alternative pathways (cyclotron irradiation of ^226^Ra, high-energy proton spallation of ^232^Th, photonuclear reactions on ^226^Ra) remain preclinical. In addition, the daughter radionuclides (^221^Fr, ^217^At, ^213^Bi) produced at the decay site remain a major dosimetric and toxicological concern, particularly for renal and salivary gland exposure (Fig. 4).^58^

**Fig. 4:**
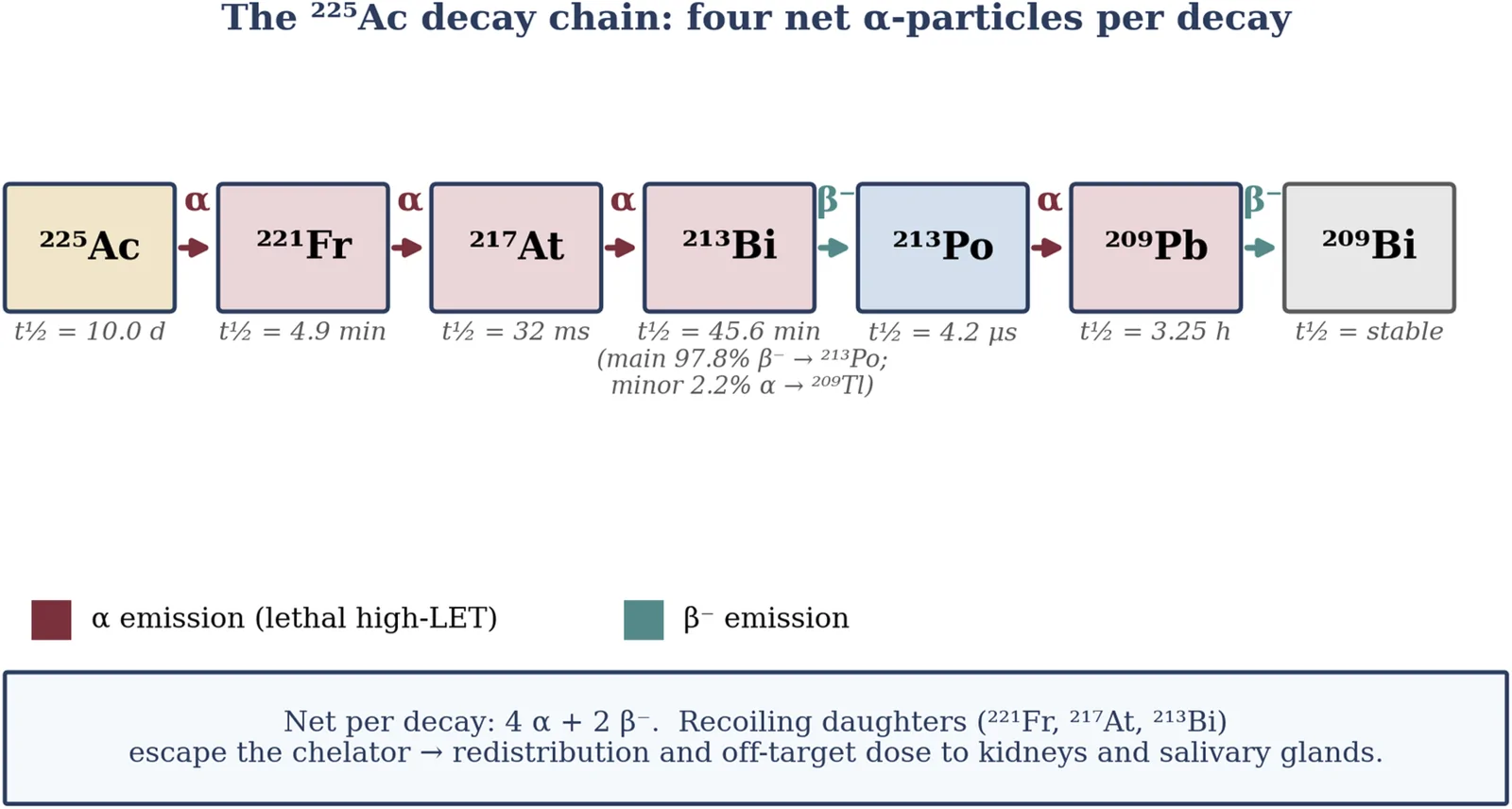
The decay chain of ^225^Ac and the daughter-redistribution problem. Decay of ^225^Ac proceeds through ^221^Fr, ^217^At, ^213^Bi, ^213^Po and ^209^Pb to stable ^209^Bi, releasing four net α-particles and two β^−^-particles per decay (the ^213^Bi step branches: ∼97.8% β^−^ to ^213^Po and ∼2.2% α to ^209^Tl). The power of ^225^Ac is due to the large cumulative α-emission, which results in energetic recoil of the daughters (^221^Fr, ^217^At, ^213^Bi) from the chelator, leading to redistribution and dose to off-target organs, such as kidneys and salivary glands. The physical half-lives are shown for each nuclide. Original schematic, drawn by the authors.

The chemical root of the daughter-redistribution problem is that none of the four alpha-decay recoil products in the ^225^Ac chain, ^221^Fr, ^217^At, ^213^Bi and ^209^Pb, share Ac^3+^'s coordination preferences, so even a chelator that fully retains the parent ^225^Ac is chemically incapable of retaining its daughters once formed *in situ*: each α-decay imparts substantial recoil energy (tens of keV) that ejects the daughter from the chelator cage regardless of binding constant. Mitigation strategies therefore target the consequence rather than the cause: co-encapsulation in nanoparticle or liposomal carriers to physically contain recoiling daughters, *in vivo* generator concepts that separate the therapeutic radionuclide from short-lived progeny by design, and renal-clearance-promoting pharmacokinetic tuning of the parent construct to minimize the time daughters spend in circulation before excretion. None of these approaches yet fully eliminates redistribution, and quantifying daughter-specific organ dose, rather than assuming it tracks the parent's biodistribution, remains an open dosimetric problem flagged in Section 8.1.

### 5.4 Emerging alpha emitters: ^212^Pb and ^211^At

Lead-212 (^212^Pb) is a much-discussed alpha emitter, serving as an *in vivo* source of ^212^Bi (*t*_1/2_ = 60.6 min). Generated on-demand from a ^224^Ra/^212^Pb generator, it avoids the supply constraints of ^225^Ac, and its 10.6 hours half-life suits peptide-based agents well. The AlphaMedix program (^212^Pb-DOTAMTATE) has shown promising Phase II results in GEP-NETs and is advancing under Perspective Therapeutics; separately, Sanofi has partnered with Orano Med on ^212^Pb-based therapies.^53^

Astatine-211 (*t*_1/2_ = 7.2 h) avoids daughter-redistribution concerns and, being chemically halogen-like (similar to iodine), is readily conjugated to biomolecules by direct covalent labeling. Clinical investigation is ongoing for brain tumors (glioblastoma) and ovarian cancer, though its short half-life and cyclotron dependency remain practical constraints.^59^

Terbium-161 (^161^Tb) is a ‘Swiss army knife’ radionuclide, emitting low-energy beta particles, conversion electrons, Auger electrons, and imageable gamma photons within a single decay scheme. Its Auger emissions cause extremely localized, high-dose damage once internalized into the cell nucleus, combining beta-particle and Auger-electron advantages. First-in-human ^161^Tb-DOTATOC studies in SSTR-positive NET patients show favorable biodistribution and dosimetry, with further clinical trials anticipated (Baum *et al.*, 2021).

Sections 3–5 above concern radioligands that are either regulatory-approved or supported by randomised phase III data; the agents surveyed in Section 6 below, by contrast, are without exception early-clinical or preclinical, and the strength of evidence for each is stated explicitly at first mention (compassionate-use series, single-arm phase I/II data, or xenograft data only). This demarcation is intentional: several of these targets (CXCR4, GRPR, mesothelin, CAIX) have single-digit-to-low-double-digit patient numbers reported to date, and none has yet produced randomised comparative data against standard of care; they should be read as hypothesis-generating rather than practice-changing.

## 6. Emerging molecular targets and novel radioligands

### 6.1 Fibroblast activation protein (FAP)-Targeted theranostics

Fibroblast activation protein (FAP), a type II transmembrane serine protease, is selectively expressed on cancer-associated fibroblasts (CAFs) in over 90% of epithelial carcinomas but shows minimal expression in normal adult tissue.^60^ This near-pan-cancer expression and favorable tumor-to-background ratio make FAP one of the most promising emerging theranostic targets.

FAP-targeted PET imaging with ^68^Ga-labeled FAP inhibitors (^68^Ga-FAPI-04, ^68^Ga-FAPI-46) has produced excellent tumor imaging across malignancies with traditionally poor ^18^F-FDG avidity, including pancreatic ductal adenocarcinoma, cholangiocarcinoma, signet-ring gastric cancer, and peritoneal carcinomatosis. Phase II/III trials of ^68^Ga-FAPI as a diagnostic agent are expected in late 2025 (NCT07217704, NCT07217717).^61^

Second-generation FAP ligands with improved pharmacokinetics and tumor residence are entering therapeutic use. The LuMIERE trial is evaluating ^177^Lu-FAP-2286 therapy, with patients selected by ^68^Ga-FAP-2286 PET/CT a true theranostic pairing using the same ligand for diagnosis and therapy. Other FAP-targeted therapeutics include ^225^Ac-FAPI and the dimeric inhibitor ^177^Lu-DOTAGA-Glu(FAPi)_2_.^62^

### 6.2 CXCR4-targeted radioligands

C-X-C motif chemokine receptor 4 (CXCR4) is highly expressed in hematological malignancies (multiple myeloma, non-Hodgkin lymphoma, AML, CLL) and some solid tumors. Early clinical and compassionate-use studies of the theranostic pair ^68^Ga-Pentixafor (diagnostic) and ^177^Lu-/^90^Y-Pentixather (therapeutic) show feasibility and initial efficacy in relapsed/refractory hematological malignancies, with myelosuppression as the main dose-limiting toxicity.^63^

### 6.3 Gastrin-releasing peptide receptor (GRPR)-Targeted agents

Gastrin-releasing peptide receptor (GRPR, bombesin receptor subtype 2) is overexpressed in prostate, breast, small-cell lung, and gastrointestinal stromal tumors. Notably, GRPR complements PSMA in prostate cancer being highly expressed in primary/early disease, where PSMA is low thereby potentially covering PSMA-negative tumors. Therapeutic (^177^Lu-RM2, ^177^Lu-NeoBOMB1) and diagnostic (^68^Ga-RM2, ^68^Ga-NeoBOMB1) GRPR agents are in early-phase clinical development.^64^

### 6.4 HER2-targeted radiopharmaceuticals

HER2 is overexpressed in ∼15–20% of breast cancers and subsets of gastric, esophageal, and other cancers. ^68^Ga- or ^89^Zr-labeled anti-HER2 antibodies/nanobodies enable non-invasive, real-time PET assessment of HER2 status, avoiding biopsy-based limitations. To circumvent the long half-life and hematologic toxicity of directly radiolabeled intact antibodies, pretargeted radioimmunotherapy using bispecific antibodies against HER2 and a radiolabeled hapten is under investigation; preclinical studies show ^225^Ac-pretargeted alpha-RIT is feasible in HER2-expressing breast cancer models.^65^

### 6.5 Radioimmunotherapy: past, present, and future

Radioimmunotherapy (RIT) therapeutic radionuclides bound to monoclonal antibodies predates the modern theranostic era and is resurging thanks to improved antibody engineering and alpha-emitting radionuclides. First-generation agents ^90^Y-ibritumomab tiuxetan (Zevalin®) and ^131^I-tositumomab (Bexxar®), both targeting CD20 in non-Hodgkin lymphoma, achieved high response rates (80–95% ORR) but were commercially unsuccessful due to rituximab competition and logistical challenges.^66^

Next-generation RIT strategies instead use pretargeting, antibody fragments (nanobodies, single-domain antibodies, minibodies), and alpha-emitting payloads. In pretargeted RIT, a bispecific antibody first binds the tumor antigen and a hapten; a radiolabeled hapten is then injected after the antibody clears, binding the pretargeted antibody at the tumor surface. This markedly improves tumor-to-normal-tissue ratios and reduces bone-marrow irradiation. Antibody conjugates bearing alpha emitters, such as the PSMA-targeting BAY 2315497, represent another novel approach.^67^

Table 3 lists additional molecular targets extending the theranostic concept beyond PSMA and SSTR.^68^ Beyond FAP, CXCR4, GRPR, and HER2 (above), mesothelin and carbonic anhydrase IX (CAIX) are notable CAIX, expressed in >95% of clear-cell renal cell carcinomas, is being tested with ^177^Lu-girentuximab in the phase 1b/2 (STARLITE-1) and phase 2 (STARLITE-2) trials. Most of these agents pair a ^68^Ga- or ^89^Zr-labeled diagnostic with a β^−^- or α-emitting therapeutic, preserving the image-and-treat theranostic principle across an expanding range of indications.

## 7. Combination strategies: synergizing radioligand therapy with systemic agents

### 7.1 RLT and immune checkpoint inhibitors

Combining radioligand therapy (RLT) with immune checkpoint inhibitors (ICIs) is among the most scientifically intriguing combination strategies. TRT induces immunogenic cell death that releases tumor neoantigens and DAMPs, upregulates PD-L1, promotes dendritic cell maturation, and can shift ‘cold’ tumors to ‘hot’. Unlike EBRT, which treats a single site, TRT simultaneously treats all PSMA- or SSTR-expressing metastatic deposits, potentially producing a broader systemic immune response (Fig. 5).^69^

**Fig. 5:**
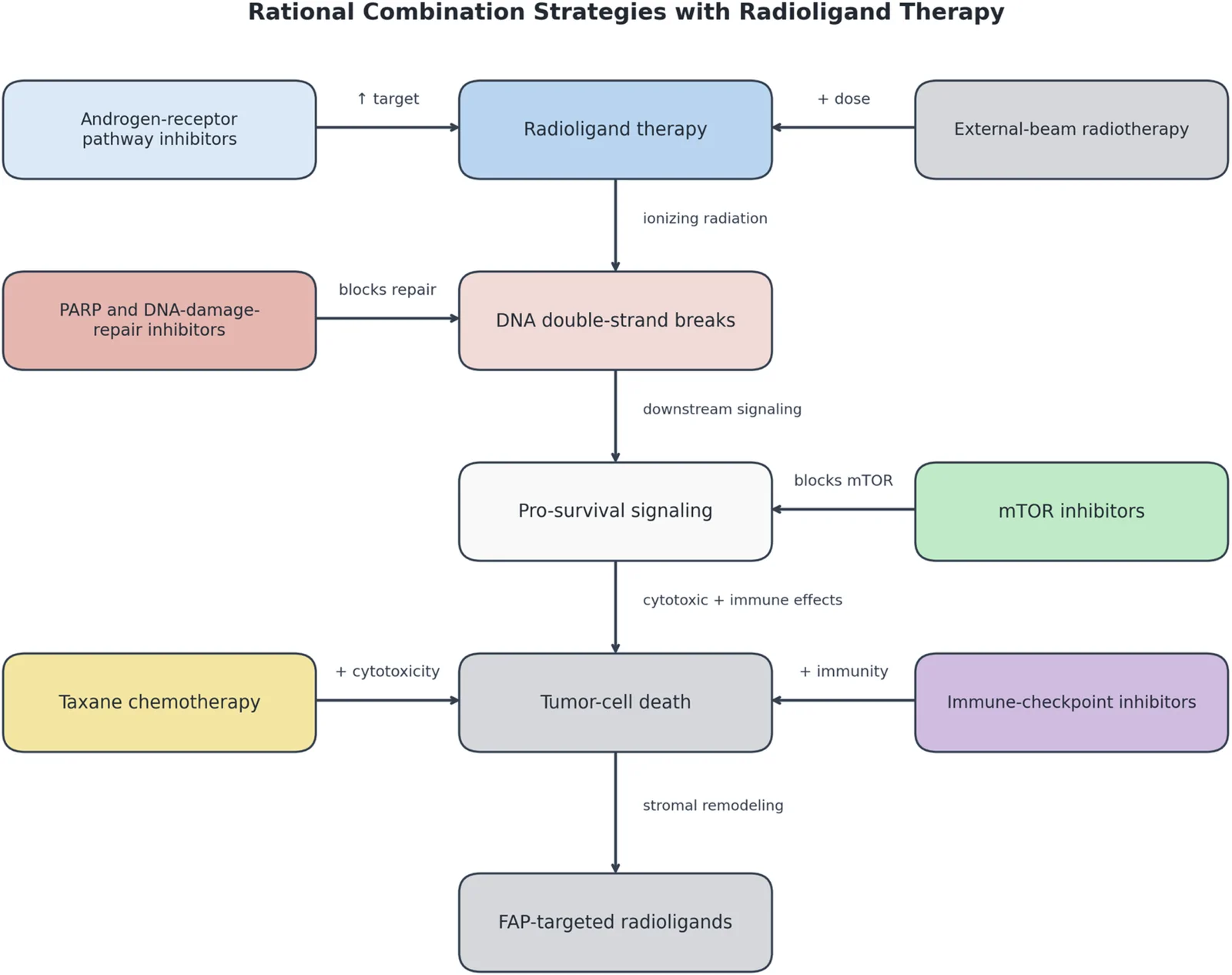
Rational combination strategies with radioligand therapy. Ionizing radiation from radioligand therapy (β^−^/α-emitter, *e.g.*^177^Lu, ^225^Ac) causes DNA double-strand breaks and activates downstream survival signaling (MAPK, PI3K/AKT/mTOR); this axis can be combined with androgen-receptor pathway inhibitors, external-beam radiotherapy, PARP/DNA-damage-repair inhibitors, and mTOR inhibitors. The resulting tumor-cell death releases tumor antigens, an effect that can be amplified by taxane chemotherapy and immune checkpoint inhibitors (anti-PD-1/PD-L1, anti-CTLA-4); FAP-targeted radioligands act in parallel by directly depleting cancer-associated fibroblasts (CAF) to remodel the tumor stroma.

Multiple trials are exploring RLT-ICI combinations. In the PRINCE trial (NCT03658447), ^177^Lu-PSMA-617 plus pembrolizumab in mCRPC showed preliminary PSA response rates superior to historical monotherapy data, though optimal sequencing, dosing, and ICI partner selection remain undetermined. A key concern is RLT-induced lymphopenia/immunosuppression counteracting ICI benefit, since beta-emitting RLT's crossfire and bystander effects can damage tumor-infiltrating lymphocytes (TILs). Proposed mitigations include dose fractionation, sequencing ICI before RLT to expand the TIL pool first, and using short-range alpha emitters that may spare peritumoral immune cells.^70^

### 7.2 RLT and DNA damage repair inhibitors

RLT combined with DNA damage repair (DDR) inhibitors exploits synthetic lethality: RLT causes DNA DSBs, while DDR inhibitors such as PARP inhibitors block repair of the accompanying single-strand breaks, leaving DSBs unrepaired and increasing tumor cell killing particularly in homologous-recombination-deficient (HRD) tumors. Preclinical prostate cancer models show synergistic cytotoxicity between ^177^Lu-PSMA-617 and olaparib, now in early-phase clinical trials; ^177^Lu-DOTATATE plus olaparib is similarly under investigation in NETs. Beyond PARP inhibitors, ATR, ATM, and DNA-PKcs inhibitors are being explored as RLT radiosensitizers to extend synthetic–lethal combinations to more HRD-positive tumor types.^71^

### 7.3 RLT and androgen receptor pathway inhibitors

Combining ^177^Lu-PSMA-617 with androgen receptor pathway inhibitors (ARPIs) is especially appealing since ARPI therapy increases PSMA expression, potentially boosting radioligand uptake and efficacy. This combination is being tested first-line for mHSPC in PSMAddition (Section 3.3); the UpFrontPSMA trial is separately assessing ^177^Lu-PSMA-617 with docetaxel and ADT in hormone-naive prostate cancer.^71^

### 7.4 RLT and chemotherapy

PRRT combined with radiosensitizing chemotherapy (the CAPTEM regimen) has shown improved outcomes in GEP-NETs: retrospective and single-arm prospective studies report 30–45% ORR for ^177^Lu-DOTATATE + CAPTEM *versus* 18% for ^177^Lu-DOTATATE monotherapy in NETTER-1, suggesting synergy. Temozolomide (alkylating) and capecitabine (antimetabolite) likely radiosensitize by inhibiting DNA repair.^72^ Randomized trials comparing PRRT + CAPTEM to PRRT alone are still needed to confirm this benefit.

## 8. Personalized dosimetry and the role of artificial intelligence

### 8.1 Current state of theranostic dosimetry

Dosimetry measuring and calculating absorbed radiation dose to tumors and organs at risk (OARs) is essential for individualized TRT planning. Unlike EBRT, TRT dosimetry has traditionally relied on fixed-activity or weight/body-surface-area dosing, a ‘one size fits all’ approach that ignores substantial interpatient variability in pharmacokinetics, biodistribution, and dosimetry.^73^

Dosimetry is inherent to the theranostic paradigm: the diagnostic scan (*e.g.*, ^68^Ga-PSMA or ^68^Ga-DOTATATE PET/CT) estimates tumor receptor density and predicted radioligand uptake, while post-therapy SPECT/CT (using ^177^Lu's gamma emissions) measures absorbed dose to tumor and OARs, *via* the Medical Internal Radiation Dose (MIRD) formalism based on time-integrated activity from serial imaging.^74^

Evidence increasingly supports dosimetry's value: absorbed tumor dose correlates with response, and kidney/bone-marrow dose with toxicity. Routine clinical adoption, however, is hindered by the need for multiple imaging time points (typically 3–5 SPECT/CT scans over 1–7 days), the time and expertise required for manual dose calculation, and the lack of standardized procedures or commercial dosimetry software.^75^

### 8.2 Artificial intelligence in theranostic dosimetry

Artificial intelligence (AI) and deep learning (DL) are now addressing these bottlenecks across the dosimetry workflow: (1) automated organ/tumor segmentation on CT and SPECT, replacing time-consuming manual contouring; (2) single-time-point dosimetry, where DL predicts the time–activity curve from one post-therapy scan; (3) PET-based dose prediction, estimating therapeutic dose distribution from diagnostic PET uptake; and (4) voxel-level dose estimation, yielding high-resolution maps of intratumoral dose variability.^76^

Convolutional neural networks (CNNs) now achieve organ-segmentation accuracy near expert manual segmentation (Dice >0.90) for kidneys, liver, and spleen, cutting segmentation time from hours to seconds on SPECT/CT.^77,78^ U-Net architectures enable fully automated dosimetry pipelines for PSMA and SSTR PET/CT tumor segmentation, while generative adversarial networks (GANs) and transformer models are being developed to predict high-resolution dose maps from low-count or single-time-point SPECT data, potentially enabling point-of-care ‘real-time’ dosimetry.^79^

AI may ultimately enable closed-loop radiotheranostic systems, linking target discovery, ligand design, imaging interpretation, dosimetry, treatment planning, and outcome prediction into a self-optimizing precision-oncology platform. Realizing this will require extensive validation, regulatory approval, and curated multi-institutional training datasets.^36^

These translational barriers remain incompletely addressed. Most AI dosimetry models to date have been developed and validated on small, retrospectively curated, single- or few-institution cohorts,^76^ so the performance reported in these settings may not generalize to the heterogeneous patient populations, acquisition protocols, and reconstruction settings encountered in routine multi-center practice. Cross-scanner and cross-protocol variability is a particular obstacle for SPECT/CT-based dosimetry, where differences in collimator choice, reconstruction algorithm, attenuation/scatter correction, and calibration factor across vendors and sites can shift quantitative activity estimates by a clinically meaningful margin, and few AI models have been harmonized or re-validated across these conditions (Kesner *et al.*, 2025). Regulatory approval is similarly nascent: no AI-based dosimetry tool has yet achieved full regulatory clearance as a standalone clinical decision-support device for radioligand therapy, and the pathway to approval will likely require prospective, multi-institutional validation against ground-truth dosimetry rather than retrospective benchmarking alone (Hofman *et al.*, 2020). Until these gaps are closed, AI-based dosimetry should be regarded as an investigational adjunct rather than a substitute for conventional dosimetric workflows.

## 9. Current challenges and limitations

### 9.1 Heterogeneous target expression and resistance mechanisms

Intra- and inter-lesional heterogeneity of target expression is a fundamental TRT challenge. Up to 10–30% of mCRPC cases show PET-negative or PET-low PSMA status and cannot be targeted by PSMA-guided RLT;^21^ discordant disease (mixed PSMA-positive/negative lesions within one patient) can allow PSMA-negative lesions to drive progression during therapy. NETs show an analogous problem, as dedifferentiation to SSTR-negative, high-grade neuroendocrine carcinoma reduces PRRT efficacy.^37^

RLT resistance is an increasingly recognized clinical problem whose mechanisms remain incompletely understood, potentially including target downregulation/silencing, upregulated DNA repair, anti-apoptotic signaling, altered radioligand pharmacokinetics (reduced uptake, increased efflux), or selection of intrinsically resistant subpopulations. Combination targeting of multiple receptors (*e.g.*, PSMA and GRPR) or pan-cancer targets such as FAP is being studied to overcome this heterogeneity.^80^

Mechanistically, the path from target expression to therapeutic response passes through several quantifiable intermediate steps that are rarely reported together in the primary literature. Membrane target density (receptor number per cell, typically 10^3^–10^5^ for PSMA and SSTR2 on responsive tumors) sets the ceiling on ligand binding at a given specific activity; receptor internalization kinetics then determine what fraction of bound activity is retained intracellularly rather than washed out, and internalizing receptors (PSMA, SSTR2, GRPR) generally support higher tumor retention than non-internalizing ones. Tumor heterogeneity superimposes a spatial dimension: because absorbed dose is delivered voxel-by-voxel, a tumor with a bimodal expression distribution can show an acceptable mean SUV on PET while a substantial low-expressing subpopulation receives a sub-therapeutic dose and drives subsequent regrowth, a mechanism increasingly invoked to explain the gap between high radiographic response rates and more modest progression-free survival in PSMA- and SSTR-RLT. Quantitative dose–response modeling that links pretherapy PET-derived receptor density, single- or multi-time-point dosimetry, and voxel-level outcome (Section 8) is the main tool currently available to close this mechanistic gap, but such modeling is not yet standard in clinical practice outside specialist centers.

### 9.2 Dose-limiting toxicities

TRT toxicities mainly reflect target expression in normal tissue and radioligand excretion routes. Xerostomia, from PSMA expression in salivary gland epithelium, is the most characteristic PSMA-RLT adverse effect usually low-grade (1–2) but capable of significantly impairing quality of life. Myelosuppression (anemia, thrombocytopenia, lymphopenia) is a dose-limiting class effect, arising from both circulating-radioligand and bone-metastasis irradiation of marrow.

Renal toxicity has been a PRRT concern, since peptides are reabsorbed in the proximal tubule *via* the megalin/cubilin system; however, co-infusing nephroprotective amino acids with ^177^Lu-DOTATATE kept clinically significant renal toxicity rare (<1% grade 3–4) in NETTER-1. Rare but severe late complications myelodysplastic syndrome or AML occur in 2–5% of patients at 5 years, likely reflecting cumulative bone-marrow radiation damage.^39,81^

### 9.3 Supply chain and manufacturing challenges

Producing and distributing therapeutic radionuclides at clinical scale is logistically constrained by the physics of radioactivity. ^177^Lu is produced by irradiating enriched ^176^Yb or ^176^Lu targets in a limited number of suitable nuclear reactors worldwide; supply disruptions, such as Novartis's 2022–2023 manufacturing issues at its Ivrea, Italy plant, can directly affect patient access.^82^

^177^Lu is produced by two distinct reactor routes with materially different specific activity. The direct route, ^176^Lu(n,γ)^177^Lu, uses enriched ^176^Lu targets and a high thermal-neutron-flux reactor but yields carrier-added product because the target isotope and product share the same element, capping achievable specific activity at a level that can be limiting for high-molar-activity peptide labelling. The indirect route, ^176^Yb(n,γ)^177^Yb → ^177^Lu (β^−^ decay, *t*_1/2_ = 1.9 h), starts from an ytterbium target and separates the ^177^Lu product chemically from the parent element, giving no-carrier-added ^177^Lu of substantially higher specific activity at the cost of a more demanding radiochemical separation. ^225^Ac is produced by three competing routes, each supply-constrained differently: milking from a ^229^Th/^225^Ac generator (the historical route, limited by the small global ^229^Th stockpile referenced above); direct cyclotron irradiation of ^226^Ra *via* the ^226^Ra(p,2n)^225^Ac reaction, which avoids the ^229^Th bottleneck but requires handling gram quantities of highly radiotoxic ^226^Ra target material; and high-energy proton spallation of ^232^Th, which can produce ^225^Ac at larger scale but co-produces the long-lived, chemically inseparable contaminant ^227^Ac that must be kept below strict limits for clinical release.

Supply constraints are even more severe for alpha emitters: global ^225^Ac supply (∼63 GBq per year from ^229^Th generators) is orders of magnitude below estimated clinical demand. Alternative pathways cyclotron irradiation of ^226^Ra, high-energy proton spallation of ^232^Th, neutron irradiation of ^226^Ra have not yet reached reliable clinical-scale production. Generator-based ^212^Pb production from ^224^Ra offers a possible on-demand solution at clinical sites.^40^

### 9.4 Regulatory and health economic considerations

Radiopharmaceutical theranostics face distinct regulatory considerations *versus* standard pharmaceuticals: companion-diagnostic imaging for patient selection, specialized nuclear-medicine/hot-lab infrastructure, radiation safety, waste management, and short product shelf life all favor decentralized manufacturing and streamlined regulatory pathways.^83^ High treatment cost also raises access concerns, particularly in low- and middle-income countries where cancer burden is high, and even in the US, where a full six-cycle Pluvicto® course costs roughly $180,000.^84^ Cost-effectiveness analyses of ^177^Lu-PSMA-617 have been inconsistent across healthcare systems, comparators, and willingness-to-pay thresholds, pointing to a need for value-based pricing and risk-sharing agreements.^85^

## 10. Future directions and perspectives

### 10.1 Expansion to new cancer types

Although RLT's clinical use has so far centered on prostate cancer and NETs, the theranostic paradigm is poised to expand well beyond these.^86^ FAP's broad expression in CAFs supports a pan-cancer approach spanning pancreatic, colorectal, breast, lung, and head-and-neck cancers; ^177^Lu-girentuximab is advancing in clear-cell renal cell carcinoma, where CAIX is expressed in >95% of tumors; and DLL3-targeted agents are in development for SCLC and high-grade neuroendocrine carcinoma. Emerging targets in glioblastoma (GD2, EGFR, integrin), ovarian cancer (mesothelin, folate receptor), and hepatocellular carcinoma (GPC3) will further extend the field.^87^

### 10.2 Multi-receptor targeting and cocktail approaches

Multi-receptor targeting strategies address target heterogeneity through: (1) cocktail approaches combining two radioligands against different receptors (*e.g.*, ^177^Lu-PSMA-617 + ^177^Lu-RM2 for dual PSMA/GRPR targeting); (2) bispecific radioligands with two binding sites; and (3) sequential alternation between radioligands to avoid target downregulation.^88^ Preclinical models show dual-targeting produces more uniform tumor irradiation and better response than single-target RLT.^89^

### 10.3 Nanoparticle-based radiopharmaceuticals

Nanoparticle platforms offer unique delivery advantages: multiple radionuclides per particle (dose amplification per targeting event), combined diagnostic/therapeutic isotopes in one construct, enhanced tumor uptake *via* the enhanced permeability and retention (EPR) effect, and potential confinement of alpha-emitting daughter radionuclides within the particle matrix eliminating ^225^Ac's daughter-redistribution problem. Liposomes, polymeric nanoparticles, inorganic nanoparticles (gold, iron oxide, silica), and dendrimers are all under study for improved TRT therapeutic index.^81,88^

### 10.4 Total-body PET and advanced imaging

Total-body PET scanners such as uEXPLORER, offering ∼40-fold higher sensitivity and whole-body imaging in a single pass, are poised to transform theranostic imaging enabling ultra-low-dose imaging, dynamic whole-body pharmacokinetic analysis, and delayed imaging (24–72 h post-injection) directly applicable to improved dosimetry and therapy planning. Parametric imaging of radioligand binding kinetics across all organs simultaneously could open new avenues in TRT pharmacodynamics and truly personalized dosimetry.^90^

### 10.5 The pharmaceutical industry landscape

Pharmaceutical-industry investment in radiopharmaceutical theranostics reflects strong confidence in the sector's long-term growth: cumulative acquisitions since 2018 total approximately $15 billion, led by Novartis ($3.9B for AAA, $2.1B for Endocyte, $1.0B for Mariana Oncology), Eli Lilly ($1.4B for POINT Biopharma), Bristol-Myers Squibb ($4.1B for RayzeBio), and AstraZeneca ($2.4B for Fusion Pharmaceuticals) (Fig. 6).^91^ Pluvicto® alone generated $1.04 billion in Novartis revenue in the first nine months of 2024, underscoring RLT's commercial potential.^92^ This capital is fueling expanded manufacturing, new alpha-emitter production methods, and an increasingly rich clinical pipeline a trajectory illustrated in Fig. 7's projection of the global nuclear-medicine market from 2013–2026 across the 99mTc, FDG-PET, other-diagnostic, and radiotherapeutic segments, annotated with the key regulatory milestones (^223^Ra-dichloride 2013, ^177^Lu-DOTATATE 2018, ^177^Lu-PSMA-617 2022) that drove the therapeutic segment's growth.^93^

**Fig. 6:**
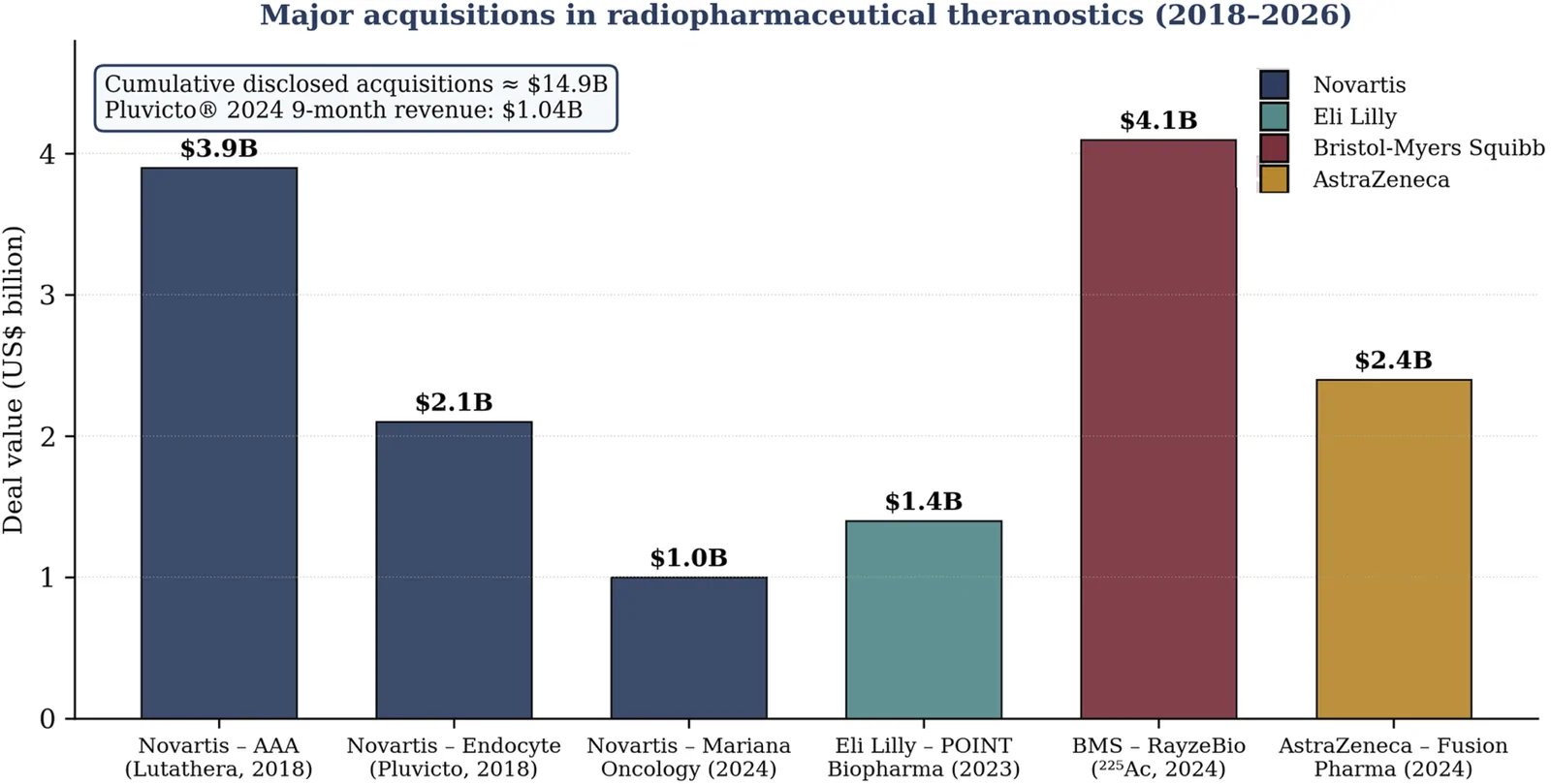
Acquisition activity in the radiopharmaceutical-theranostics sector (2018-2024).

**Fig. 7:**
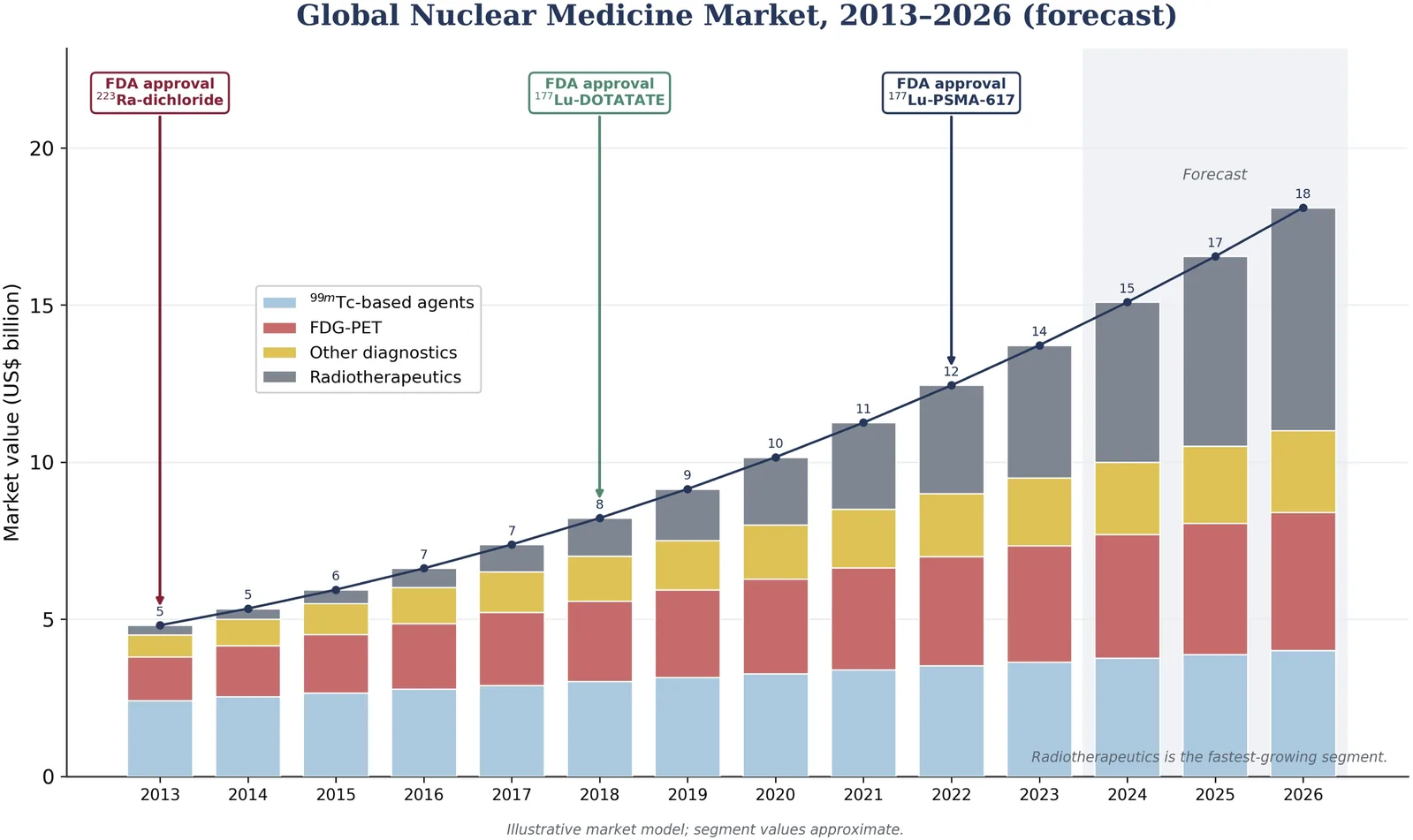
Global Nuclear Medicine Market 2013–2026 Forecast. Stacked bar chart of market growth by segment: ^99^ᵐTc-based agents (light blue), FDG-PET (red), other diagnostics (yellow), and radiotherapeutics (gray). Overlaid are key regulatory events such as FDA approval of ^223^Ra-dichloride (2013), ^177^Lu-DOTATATE (2018), and ^177^Lu-PSMA-617 (2022). The radiotherapeutics segment is the fastest growing segment. Illustrative market model created for this review; segment values are approximate and regulatory milestones reflect the FDA approval dates.

Table 4 details these acquisitions and licensing agreements from 2018–2026.^94^ Beyond the deals above, Sanofi's licensing of Orano Med's ^212^Pb assets reflects the same strategic shift: from acquiring approved β^−^-emitting products toward building ^225^Ac- and ^212^Pb-based targeted-alpha-therapy pipelines and the manufacturing capacity to support them.^95^

**Table 4:** The major pharmaceutical investments in radiopharmaceutical theranostics (2018–2026)

Acquirer	Target company	Value (USD)	Year	Key assets/Rationale
Novartis	Advanced Accelerator applications (AAA)	$3.9 B	2018	Lutathera® (^177^Lu-DOTATATE) NET PRRT platform
Novartis	Endocyte	$2.1 B	2018	^177^Lu-PSMA-617 (Pluvicto®) PSMA-RLT platform
Eli Lilly	POINT Biopharma	$1.4 B	2023	PNT2002 (^177^Lu-PSMA-I&T) Next-gen PSMA agents
Bristol-Myers Squibb	RayzeBio	$4.1 B	2024	^225^Ac-based TAT pipeline actinium production
AstraZeneca	Fusion pharmaceuticals	$2.4 B	2024	Alpha-emitter platform FPI-2265 (^225^Ac-PSMA)
Novartis	Mariana oncology	$1.0 B	2024	Novel radiopharmaceutical targets and pipeline
Sanofi	Orano Med (license)	Undisclosed	2024–25	^212^Pb-based targeted-alpha-therapy platform

## 11. Critical view and perspective on the field

This review adopts a deliberately integrative rather than encyclopedic scope. Rather than treating radiobiology, pivotal-trial outcomes, emerging molecular targets, combination pharmacology, dosimetry informatics, and the commercial pipeline as separate literature, we synthesize them into a single narrative arc that follows the theranostic paradigm from mechanism (Section 2) through regulatory approval (Sections 3–5) to the frontier of personalization (Sections 6–10). Most existing reviews in this space focus narrowly on a single radionuclide class (*e.g.*, PSMA-targeted alpha or beta emitters) or a single clinical trial (VISION, NETTER-1), and treat artificial intelligence-based dosimetry and industry investment trends, when covered at all, as an afterthought rather than as evidence of where the field is structurally heading. By explicitly connecting trial-level efficacy data to the biophysical rationale that produced it, and by placing AI dosimetry and pharmaceutical-industry acquisition activity alongside clinical results, this review aims to give readers a working model of not just what has been approved, but why the field is moving toward alpha-emitters, combination regimens, and image-based personalized dosing simultaneously.

The depth of coverage is narrative and qualitative rather than systematic: we did not perform a PRISMA-style systematic search, meta-analysis, or formal risk-of-bias assessment, and the review should not be read as a substitute for one. Coverage is weighted toward late-phase and regulatory-relevant evidence (VISION, NETTER-1, PSMAfore/PSMAddition, ALSYMPCA) and toward mechanistic and translational literature published through 2026; earlier foundational work on radioiodine and somatostatin receptor imaging is covered only to the extent needed to establish context. Preclinical and early-phase (Phase I/II) data on emerging targets such as FAP, GRPR, and HER2 are discussed to indicate direction of travel, not as established standards of care, and are flagged as such throughout.

Where this review differs most from prior surveys is in its treatment of combination strategies and dosimetry as a single, connected problem rather than two separate ones: the rationale for combining RLT with DNA-damage-repair inhibitors, checkpoint blockade, or androgen-deprivation therapy is, in our view, inseparable from the dosimetric and toxicity constraints that AI-based personalization is now being built to solve. We also make explicit an observation that is often left implicit in the literature: the acceleration of pharmaceutical-industry acquisitions in this space since 2018 is itself evidence that the underlying radiobiology and early trial signals are considered durable by capital markets, not merely academically promising. Readers should treat the market-landscape discussion (Section 10.5) as illustrative context for this argument, not as a rigorous financial analysis.

To state the knowledge gap explicitly: existing reviews of this literature (*e.g.*, Bodei *et al.*, 2022; Jadvar *et al.*, 2018) are comprehensive on trial outcomes but were written before the PSMA-62/PSMA-CY313 seconds-generation ligand data, the NETTER-2 first-line result, and the RayzeBio/Fusion/Mariana acquisition wave summarized in Table 4 were available, and none integrates radiochemical mechanism (chelator/linker design, Section 2.4) with clinical outcome and dosimetric/AI infrastructure within a single narrative arc. That integration, rather than any single new clinical finding, is this review's contribution to the literature.

The principal benefit of this integrative approach to current knowledge of the field is a decision-relevant synthesis rather than a restatement of individual findings. By mapping clinical trial outcomes, radiobiological rationale, dosimetric constraints, and commercial momentum onto a single framework, this review aims to help clinicians and trialists judge which combination strategies and alpha-emitting agents are best positioned for near-term randomized testing, help medical physicists and AI developers anticipate which dosimetry tools most urgently warrant prospective validation, and give the wider field an evidence-based rationale for why radiopharmaceutical theranostics is likely to expand well beyond its current prostate cancer and neuroendocrine tumor indications.

## 12. Conclusions

Radiopharmaceutical theranostics has grown from a nuclear-medicine subspecialty into a cornerstone of precision oncology, transforming prostate cancer and neuroendocrine tumor therapy with clear potential for wider impact. From VISION and NETTER-1 through earlier-line trials (PSMAfore, PSMAddition, NETTER-2), a strong evidence base now supports radioligand therapy as standard of care. Targeted alpha therapy is the natural next step: ^223^Ra is approved, while ^225^Ac, ^212^Pb and ^211^At advance through clinical development, offering high-LET, oxygen-independent, largely irreparable DNA damage that can overcome beta-emitter resistance and better address micrometastatic disease though radionuclide supply, daughter redistribution, and dose-limiting toxicities remain to be solved before widespread TAT adoption. Beyond PSMA and SSTR, emerging targets FAP, CXCR4, GRPR, HER2, CAIX, and DLL3 are poised to extend theranostics across the oncology space; FAP's near-universal stromal expression is especially notable for a potential pan-cancer approach. Combining RLT with immunotherapy, PARP inhibitors, and ARPIs is scientifically well-grounded and clinically promising, though optimal sequencing and patient selection still require well-designed randomized trials. AI-enabled personalized dosimetry and total-body PET will be central to moving from empiric fixed dosing toward truly individualized, dosimetry-guided treatment. Approximately $15 billion in pharmaceutical acquisitions since 2018 reflects industry confidence in this trajectory and is fueling new manufacturing capacity, alpha-emitter production methods, and clinical pipelines. Realizing the field's full potential will require sustained collaboration among nuclear medicine physicians, oncologists, radiochemists, medical physicists, regulators, and industry partners. The next decade should bring truly personalized, molecularly targeted cancer treatment within reach for the majority of common cancers.

## Author contributions

Humaira Zulfiqar: conceptualization, validation, visualization, writing – review & editing. Muhammad Waqas: writing – review & editing. Ijaz Ali: writing – original draft, writing – review & editing.

## Conflicts of interest

The authors have no conflicts of interest to declare.

## Data Availability

This is a review article. No primary data were collected. Data presented are all published data mentioned in the cited literature.
